# Supplementary material for: Deriving fine-scale models of human mobility from aggregated origin-destination flow data
Source: PLoS Comput Biol. 2021 Feb 11;17(2):e1008588. doi: 10.1371/journal.pcbi.1008588 (PMC7920350; doi:10.1371/journal.pcbi.1008588)
Supplement: S1 Text — More in-depth description of the models discussed in the main text (GM and RM1-RM4) together with additional radiation models implemented as part of this project. Additional tables for parameter interpretation, goodness of fit of all implemented models, and parameter estimates for all implemented models. (PDF) [file pcbi.1008588.s001.pdf]

Deriving Fine-Scale Models of Human Mobility from Aggregated Origin-Destination Flow Data
-
S1 Text: Expanded methods and results

Constanze Ciavarella, Neil Ferguson

Contents

1 Mathematical models of human mobility **1**

2 Parameter interpretation **3**

3 Model fits by country **5**

4 Parameter estimates by model **8**

1 Mathematical models of human mobility

One objective of this project was to compare the performance of the gravity and radiation models.

Let i and j be locations at a distance d_{ij} from each other. Denote with m_i the population of location i. We assume the flow GM_{ij} from location i to location j to be approximated by:

$$(GM) \qquad 10^{\kappa} \frac{m_i^{\alpha} m_j^{\beta}}{1 + \left(\frac{d_{ij}}{10^{\gamma}}\right)^{\varepsilon}}.$$

The symmetrised version we used with the Kenyan dataset is:

$$(GM') \qquad 10^{\kappa - \gamma\varepsilon} \frac{\left(m_i^{\alpha} m_j^{\beta} + m_j^{\alpha} m_i^{\beta}\right)}{1 + \left(\frac{d_{ij}}{10^{\gamma}}\right)^{\varepsilon}}.$$

The two parameters of the distance kernel (in the denominator) ensure the gravity model is fitted to the distance distribution seen in the data.

Radiation models do not take distance into account at all; indeed, the only information that goes into the model is the size of the origin population as well as the relative proximity and size of possible destinations. We implemented the original formulation and two variations of radiation model that are adapted to modelling journeys between overnight locations and hence decrease the probability of short-distance trips:

RM v0 the radiation model as originally conceived by [1],

RM v1 a variation put forward by [2]: the origin population is multiplied by a parameter η,

RM v2 another variation by [2]: the origin population is inflated by a parameter θ.

Mathematically, these formulations can be written as

$$\begin{aligned}
&(RMv0) \qquad T_i \, \frac{m_i \, m_j}{(m_i + r_{ij} + m_j)\,(m_i + r_{ij})},\\
&(RMv1) \qquad T_i \, \frac{\eta \, m_i \, m_j}{(\eta \, m_i + r_{ij} + m_j)(\eta \, m_i + r_{ij})},\\
&(RMv2) \qquad T_i \, \frac{(m_i + \theta)\, m_j}{(m_i + \theta + r_{ij} + m_j)(m_i + \theta + r_{ij})}.
\end{aligned}$$

The corresponding symmetrised equations for Kenya are:

$$\begin{aligned}
&(RMv0') \qquad \left(T_i \, \frac{m_i \, m_j}{(m_i + r_{ij} + m_j)\,(m_i + r_{ij})} + T_j \, \frac{m_j \, m_i}{(m_j + r_{ji} + m_i)\,(m_j + r_{ji})} \right),\\
&(RMv1') \qquad \left(T_i \, \frac{\eta \, m_i m_j}{(\eta \, m_i + r_{ij} + m_j)(\eta \, m_i + r_{ij})} + T_j \, \frac{\eta \, m_j m_i}{(\eta \, m_j + r_{ji} + m_i)(\eta \, m_j + r_{ji}))} \right),\\
&(RMv2') \qquad \left(T_i \, \frac{(m_i + \theta)\, m_j}{(m_i + \theta + r_{ij} + m_j)(m_i + \theta + r_{ij})} + T_j \, \frac{(m_j + \theta)\, m_i}{(m_j + \theta + r_{ji} + m_i)(m_j + \theta + r_{ji})} \right).
\end{aligned}$$

The numbers T_i counting the residents travelling to a destination outside their origin location are not always available. Radiation models do not say how many people travel overall, just the distribution of destination among journeys. Since we have no information on the number of travellers per administrative unit, we resort to modelling the number of travellers separately, Let $\{c_i\}$ bet the set of fine scale cells associated with administrative unit i. We devised different formulations to estimate T_{c_i} using one or two parameters to be fit alongside the other model parameters:

$$\begin{aligned}
&(t0) \qquad T_{c_i} = 10^{\kappa}\\
&(t1) \qquad T_{c_i} = 10^{\kappa} \sum_{j \neq i} n_{ij}\\
&(t2) \qquad T_{c_i} = 10^{\kappa} \, m_i^{\alpha}\\
&(t3) \qquad T_{c_i} = 10^{\kappa} \, m_{c_i}^{\alpha}\\
&(t4) \qquad T_{c_i} = 10^{\kappa} \, m_{c_i}\\
&(t5) \qquad T_{c_i} = \kappa \, m_{c_i}
\end{aligned}$$

where n_{ij} is the number of trips with origin i and destination j. Note that (t2) and (t3) coincide at the administrative unit level. Note also that (t1) Note also that we implemented (t1) only for Namibia, since the origin-destination matrix was non-directional for Kenya.

We implemented most of the possible combinations of radiation models with different estimates for the number of travellers. The best radiation model over the administrative unit and 20km scales and for both datasets was model RM v2 t3 (called RM4 in the main text). We hence decided to limit further fine-scale simulations of the radiation models to the best model RM v2 t3, the most simple model RM v0 t5 (called RM1 in the main text), as well as models RM v0 t3 and RM v2 t4 (respectively RM2 and RM3 in the main text) that represent some sort of intermediate models between the first two. These four models have been discussed further in the main text.

2 Parameter interpretation

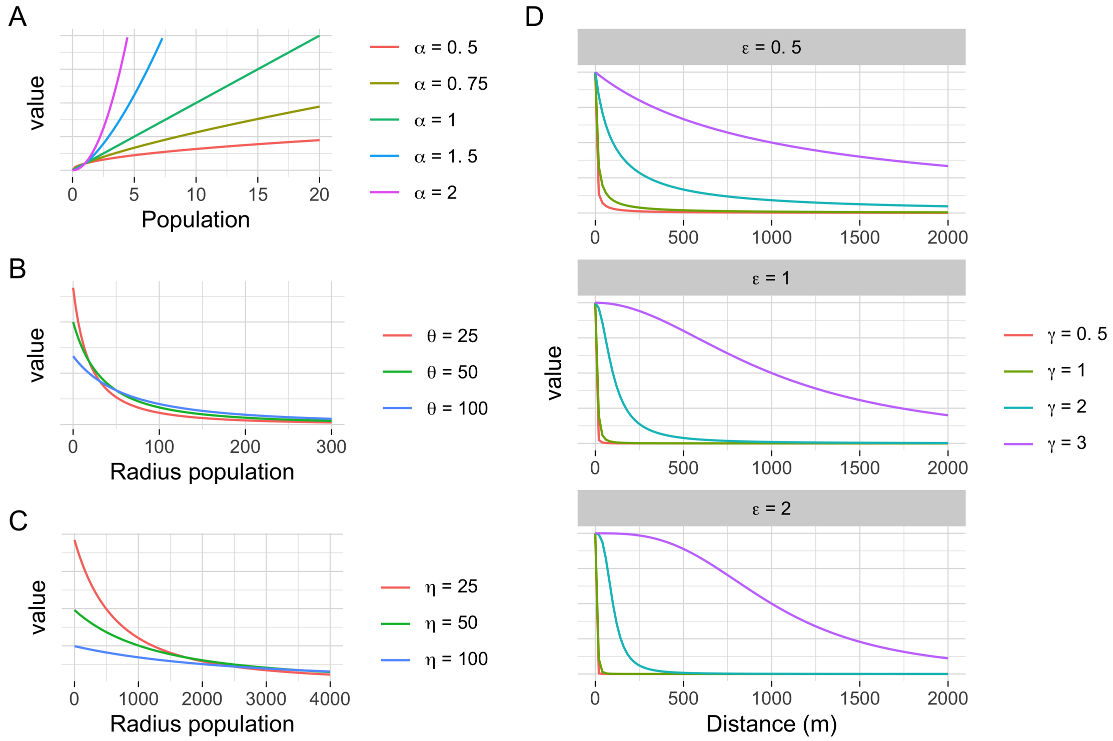

Fig 1. Elementary function plots for the interpretation of model parameter estimates. (A) value = Population$^\alpha$, for $\alpha \in \{0.5, 0.75, 1, 1.5, 2\}$, (B) value = $\theta / \big((\theta + \text{Radius population} + \text{Destination population}) \cdot (\theta + \text{Radius population})\big)$, for $\theta \in \{25, 50, 100\}$ and with arbitrary constant destination population = 50, (C) value = $1/(1 + (\frac{Distance}{10^\gamma})^\varepsilon)$, for $\gamma \in \{0.5, 1, 2, 3\}$ and $\varepsilon \in \{0.5, 1, 2\}$.

Table 1. Interpretation of model parameters.

Parameter	Formula	Role	Interpretation
disp	log-likelihood	over-dispersion parameter of negative binomial distribution	If $disp = 0$, the negative binomial coincides with the Poisson distribution. Over-dispersion in the data is indicated by $disp > 0$: the higher the value of *disp* the more over-dispersion there is.
κ	GM, RM	proportionality constant	Fitting κ ensures the model can reproduce the overall volume of flows seen in each dataset.
α	GM, RM	exponent of origin population	If $\alpha = 1$, travel propensity is proportional to the size of the origin population; if $\alpha < 1$, travel propensity is proportionally smaller for larger origin populations; if $\alpha > 1$, travel propensity is proportionally larger for larger origin populations.
β	GM	exponent of destination population	If $beta = 1$, destination locations attract a number of travellers proportional to their population; if $\beta < 1$, high population locations attract a proportionally lower number of travellers; if $\beta > 1$, high population locations attract a proportionally higher number of travellers.
γ	spatial kernel of GM	distance scale	If $\gamma < 2$, trips of more than 100m are discounted by more than half; if $\gamma < 3$, trips of more than 1km are discounted by more than half.
ε	spatial kernel of GM	exponent of scaled distance	The higher the value of ε the steeper travel propensity falls with distance.
θ	RM-v2	increment of origin population	Lower values of θ yield fewer long-distance trips because travellers are absorbed by an earlier destination; higher values of θ provide travellers with a higher energy level and hence yield proportionally more long-distance trips.
η	RM-v1	factor of origin population	If $\eta < 1$, travellers have a lower energy level and hence make fewer trips where they pass numerous destinations; if $\eta > 1$, travellers have a higher energy level and hence are inclined travel past more location before reaching their destination.

3 Model fits by country

We compute additional goodness-of-fit measures on top of the log-likelihood to compare model performance. We introduce the following notation for the log-likelihoods

$$
\begin{aligned}
&(1) \quad Model\ log-likelihood && = \ell_M := log\mathscr{L}(Model|Data),\\
&(2) \quad Intercept\ modellog-likelihood && = \ell_I := log\mathscr{L}(Intercept\ model|Data),\\
&(3) \quad Saturated\ log-likelihood && = \ell_S := log\mathscr{L}(Data|Data),
\end{aligned}
$$

where the intercept model is represented by an origin-destination matrix where each flow has as value the mean of the observed flows across all origin-destination pairs. We can now define the following goodness-of-fit measures

$$
\begin{aligned}
&(4) \quad Deviance && = \ell_S - \ell_M,\\
&(5) \quad Pseudo\ R\text{-}squared && = 1 - \frac{\ell_S - \ell_M}{\ell_S - \ell_I}.
\end{aligned}
$$

We compute these log-likelihoods assuming a negative binomial distribution, employing the mean estimated overdispersion parameter *disp* for the respective model, and a Poisson distribution. The negative binomial deviance stays mostly constant, meaning that it is not able to truly assess goodness of fit. The Poisson deviance is very high and shows greater variation, confirming that the Poisson distribution is not suitable to describe flow counts. The negative binomial pseudo R-squared are generally consistent with the log-likelihood and decrease as the log-likelihood decreases. This indicates that the models manage to reproduce a good amount of the information present in the data. If we use a Poisson distribution, the pseudo R-squared values vary more dramatically but generally decrease with decreasing log-likelihood. This is due to the fact that the model and intercept log-likelihoods using the Poisson distribution are very high.

Table 2. Kenya: all mobility models Mean value and 95% credible interval (CrI) of the log-likelihood of the gravity model, GM, and all implemented radiation models for Kenya at varying spatial resolutions across all 4 MCMC chains. N.B. models RM4 and RM-v2-t2 coincide at administrative unit scale (compare formulas (t2) and (t3) in section Mathematical models of human mobility).

Scale	log-likelihood	Model	disp	Deviance (negative binomial)	Pseudo R-squared (negative binomial)	Deviance (Poisson)	Pseudo R-squared (Poisson)
Admin. unit	-44 212 (-44 216, -44 210)	RM-v2-t2	1.88	3 325	0.657	136 856 541	0.815
Admin. unit	-44 212 (-44 216, -44 210)	RM-v2-t3	1.88	3 319	0.657	135 280 067	0.818
Admin. unit	-44 221 (-44 224, -44 220)	RM-v2-t4	1.88	3 330	0.656	157 997 155	0.787
Admin. unit	-44 229 (-44 232, -44 227)	RM-v0-t3	1.89	3 329	0.654	193 032 373	0.740
Admin. unit	-44 229 (-44 231, -44 228)	RM-v0-t5	1.89	3 327	0.655	193 235 631	0.739
Admin. unit	-44 229 (-44 233, -44 227)	RM-v1-t2	1.89	3 329	0.654	181 620 023	0.755
Admin. unit	-44 229 (-44 233, -44 227)	RM-v1-t3	1.89	3 329	0.654	181 620 023	0.755
Admin. unit	-44 565 (-44 568, -44 563)	RM-v1-t0	2.09	3 326	0.618	392 623 246	0.470
Admin. unit	-44 572 (-44 575, -44 570)	RM-v2-t0	2.10	3 320	0.617	264 213 579	0.644
Admin. unit	-44 732 (-44 738, -44 730)	GM	2.20	3 330	0.598	235 272 559	0.683
20km	-43 728 (-43 732, -43 727)	RM-v2-t3	1.62	3 329	0.704	124 138 661	0.833
20km	-43 821 (-43 824, -43 819)	RM-v2-t4	1.66	3 344	0.695	240 152 821	0.676
20km	-43 865 (-43 868, -43 863)	RM-v1-t3	1.69	3 327	0.691	171 356 420	0.769
20km	-43 908 (-43 911, -43 906)	RM-v0-t3	1.71	3 335	0.687	508 765 498	0.314
20km	-43 981 (-43 984, -43 980)	RM-v0-t5	1.75	3 329	0.680	550 467 496	0.258
20km	-44 333 (-44 337, -44 331)	RM-v1-t2	1.95	3 328	0.643	1 019 600 478	-0.375
20km	-44 347 (-44 351, -44 345)	RM-v2-t2	1.96	3 321	0.642	1 578 664 301	-1.129
20km	-44 445 (-44 450, -44 442)	GM	2.02	3 320	0.632	458 848 828	0.381
20km	-44 811 (-44 814, -44 809)	RM-v1-t0	2.25	3 332	0.588	1 821 350 564	-1.457
20km	-44 811 (-44 815, -44 810)	RM-v2-t0	2.25	3 335	0.588	1 932 609 050	-1.607
10km	-43 780 (-43 783, -43 778)	RM-v2-t3	1.64	3 347	0.698	125 227 829	0.831
10km	-43 940 (-43 943, -43 938)	RM-v2-t4	1.73	3 329	0.684	493 711 112	0.334
10km	-44 024 (-44 027, -44 023)	RM-v0-t3	1.77	3 341	0.675	1 126 907 653	-0.520
10km	-44 070 (-44 073, -44 069)	RM-v0-t5	1.80	3 329	0.671	1 112 976 932	-0.501
10km	-44 309 (-44 315, -44 306)	GM	1.94	3 313	0.647	418 172 437	0.436
5km	-43 831 (-43 834, -43 829)	RM-v2-t3	1.67	24 262	-1.227	431 744 541 981	-581.309
5km	-44 050 (-44 054, -44 049)	RM-v2-t4	1.79	36 599	-2.599	106 490 517 881 022	-143 626.449
5km	-44 211 (-44 216, -44 209)	GM	1.88	3 322	0.657	434 698 710	0.414

Table 3. Namibia: all mobility models. Mean value and 95% credible interval (CrI) of the log-likelihood of the gravity model, GM, and all implemented radiation models for Namibia at varying spatial resolutions across all 4 MCMC chains. N.B. models RM4 and RM-v2-t2 coincide at administrative unit scale (compare formulas (t2) and (t3) in section Mathematical models of human mobility).

Scale	log-likelihood	Model	disp	Deviance (negative binomial)	Pseudo R-squared (negative binomial)	Deviance (Poisson)	Pseudo R-squared (Poisson)
Admin. unit	-30 683 (-30 687, -30 682)	RM-v2-t2	1.61	4 600	0.590	327 677	0.701
Admin. unit	-30 683 (-30 687, -30 682)	RM-v2-t3	1.61	4 600	0.590	328 206	0.700
Admin. unit	-30 745 (-30 748, -30 743)	RM-v2-t4	1.64	4 590	0.584	337 209	0.692
Admin. unit	-30 753 (-30 756, -30 751)	RM-v2-t1	1.64	4 599	0.583	307 843	0.719
Admin. unit	-30 821 (-30 825, -30 819)	RM-v1-t2	1.65	4 642	0.577	392 127	0.642
Admin. unit	-30 821 (-30 825, -30 819)	RM-v1-t3	1.65	4 642	0.577	392 127	0.642
Admin. unit	-30 822 (-30 825, -30 820)	RM-v1-t1	1.66	4 620	0.576	368 241	0.664
Admin. unit	-31 608 (-31 611, -31 607)	RM-v0-t3	1.99	4 685	0.492	2 843 656	-1.597
Admin. unit	-31 639 (-31 642, -31 637)	RM-v1-t0	2.01	4 674	0.488	928 866	0.152
Admin. unit	-31 784 (-31 788, -31 782)	RM-v2-t0	2.08	4 680	0.471	3 072 385	-1.806
Admin. unit	-31 857 (-31 859, -31 856)	RM-v0-t5	2.07	4 772	0.463	3 649 244	-2.333
Admin. unit	-32 158 (-32 163, -32 155)	GM	1.79	4 675	0.726	5 352 420	0.500
20km	-31 637 (-31 640, -31 635)	RM-v2-t3	2.00	4 692	0.489	451 166	0.588
20km	-31 676 (-31 679, -31 675)	RM-v2-t4	2.01	4 711	0.484	441 096	0.597
20km	-31 895 (-31 898, -31 894)	RM-v1-t1	2.11	4 731	0.458	3 606 469	-2.294
20km	-31 999 (-32 003, -31 997)	RM-v1-t2	2.15	4 757	0.446	4 066 580	-2.714
20km	-32 095 (-32 099, -32 094)	RM-v1-t3	2.20	4 757	0.434	691 257	0.369
20km	-32 543 (-32 546, -32 542)	RM-v2-t1	2.41	4 822	0.376	33 451 503	-29.551
20km	-32 573 (-32 577, -32 572)	RM-v2-t2	2.43	4 816	0.371	26 178 809	-22.909
20km	-32 696 (-32 702, -32 694)	GM	2.01	4 740	0.689	26 560 254	-1.482
20km	-32 712 (-32 716, -32 711)	RM-v1-t0	2.52	4 800	0.352	6 690 210	-5.110
20km	-32 771 (-32 775, -32 770)	RM-v0-t3	2.54	4 825	0.344	15 474 195	-13.132
20km	-32 895 (-32 898, -32 893)	RM-v2-t0	2.61	4 831	0.326	24 850 322	-21.696
20km	-34 183 (-34 186, -34 182)	RM-v0-t5	3.28	5 101	0.119	23 786 668	-20.724
10km	-31 375 (-31 379, -31 373)	RM-v2-t3	1.89	4 659	0.518	430 140	0.607
10km	-31 412 (-31 415, -31 410)	RM-v2-t4	1.91	4 654	0.514	435 083	0.603
10km	-32 634 (-32 640, -32 631)	GM	1.99	4 719	0.694	47 026 873	-3.394
10km	-32 808 (-32 811, -32 806)	RM-v0-t3	2.57	4 810	0.339	28 675 389	-25.189
10km	-34 412 (-34 415, -34 411)	RM-v0-t5	3.43	5 125	0.077	34 419 500	-30.435
5km	-31 057 (-31 061, -31 055)	RM-v2-t3	1.76	4 624	0.553	376 745	0.656
5km	-31 082 (-31 086, -31 081)	RM-v2-t4	1.77	4 630	0.550	387 644	0.646
5km	-32 539 (-32 544, -32 537)	GM	1.95	4 696	0.701	83 436 121	-6.796
5km	-32 595 (-32 598, -32 594)	RM-v0-t3	2.47	4 773	0.368	35 342 859	-31.278
5km	-34 654 (-34 657, -34 653)	RM-v0-t5	3.57	5 179	0.032	48 373 769	-43.179

4 Parameter estimates by model

Table 4. GM: Kenya and Namibia. Mean value and 95% credible interval (CrI) of fitted parameters of the gravity model, GM, for Kenya and Namibia at varying spatial resolutions across all 4 MCMC chains. Parameters shown are the proportionality constant, κ, the power on the origin population, α, the power on the destination population, β, the distance scale, γ, the spatial kernel power, ε, and the over-dispersion parameter of the negative binomial distribution, *disp*.

Country	Scale	log-likelihood	κ	α	β	γ	ε	*disp*
Kenya	Admin. unit	-44 732 (-44 738, -44 730)	3.22 (2.74, 3.69)	1.67 (1.55, 1.77)	0.58 (0.47, 0.71)	1.89 (0.09, 3.76)	2.31 (2.25, 2.37)	2.20 (2.13, 2.28)
Kenya	20km	-44 445 (-44 450, -44 442)	2.77 (2.50, 3.02)	1.53 (1.47, 1.59)	0.81 (0.76, 0.86)	1.68 (0.08, 3.35)	2.30 (2.25, 2.35)	2.02 (1.95, 2.09)
Kenya	10km	-44 309 (-44 315, -44 306)	2.67 (2.42, 2.92)	1.63 (1.56, 1.68)	0.74 (0.69, 0.79)	1.55 (0.08, 3.12)	2.29 (2.23, 2.34)	1.94 (1.87, 2.00)
Kenya	5km	-44 211 (-44 216, -44 209)	2.44 (2.20, 2.70)	1.65 (1.61, 1.70)	0.77 (0.73, 0.82)	1.36 (0.07, 2.75)	2.27 (2.23, 2.31)	1.88 (1.82, 1.94)
Namibia	Admin. unit	-32 158 (-32 163, -32 155)	-4.79 (-4.98, -4.53)	1.13 (1.09, 1.18)	1.00 (0.95, 1.04)	2.80 (2.66, 2.93)	1.08 (1.05, 1.12)	1.79 (1.74, 1.84)
Namibia	20km	-32 696 (-32 702, -32 694)	-3.06 (-3.22, -2.90)	0.99 (0.97, 1.01)	0.94 (0.92, 0.96)	2.12 (1.97, 2.27)	1.12 (1.09, 1.15)	2.01 (1.95, 2.07)
Namibia	10km	-32 634 (-32 640, -32 631)	-2.96 (-3.11, -2.81)	1.00 (0.98, 1.02)	0.99 (0.97, 1.01)	1.59 (1.41, 1.75)	1.06 (1.02, 1.09)	1.99 (1.93, 2.05)
Namibia	5km	-32 539 (-32 544, -32 537)	-2.84 (-2.98, -2.69)	1.01 (0.99, 1.03)	1.05 (1.03, 1.08)	1.35 (1.18, 1.52)	1.07 (1.04, 1.10)	1.95 (1.89, 2.01)

Table 5. RM v0 t3 (called RM2 in the main text): Kenya and Namibia. Mean value and 95% credible interval (CrI) of fitted parameters of radiation model, RM v0 t3 (RM2 in the main text), for Kenya and Namibia at varying spatial resolutions across all 4 MCMC chains. Parameters shown are the proportionality constant, κ, the power on the origin population, α, and the over-dispersion parameter of the negative binomial distribution, *disp*.

Country	Scale	log-likelihood	κ	α	*disp*
Kenya	Admin. unit	-44 229 (-44 232, -44 227)	0.86 (0.48, 1.21)	0.98 (0.92, 1.05)	1.89 (1.83, 1.96)
Kenya	20km	-43 908 (-43 911, -43 906)	2.53 (2.34, 2.72)	0.78 (0.74, 0.81)	1.71 (1.65, 1.77)
Kenya	10km	-44 024 (-44 027, -44 023)	2.79 (2.61, 2.98)	0.80 (0.76, 0.84)	1.77 (1.71, 1.84)
Kenya	5km	-44 151 (-44 154, -44 149)	3.13 (2.95, 3.31)	0.80 (0.76, 0.84)	1.84 (1.78, 1.91)
Namibia	Admin. unit	-31 608 (-31 611, -31 607)	2.74 (2.55, 2.95)	0.44 (0.39, 0.48)	1.99 (1.93, 2.05)
Namibia	20km	-32 771 (-32 775, -32 770)	4.02 (3.92, 4.11)	0.20 (0.17, 0.22)	2.54 (2.47, 2.62)
Namibia	10km	-32 808 (-32 811, -32 806)	4.11 (4.03, 4.19)	0.18 (0.15, 0.20)	2.57 (2.50, 2.65)
Namibia	5km	-32 595 (-32 598, -32 594)	4.27 (4.21, 4.34)	0.14 (0.12, 0.16)	2.47 (2.40, 2.54)

Table 6. RM v0 t5 (called RM1 in the main text): Kenya and Namibia. Mean value and 95% credible interval (CrI) of fitted parameters of radiation model, RM v0 t5 (RM1 in the main text), for Kenya and Namibia at varying spatial resolutions across all 4 MCMC chains. The parameters shown are the proportionality constant, κ, and the over-dispersion parameter of the negative binomial distribution, *disp*.

Country	Scale	log-likelihood	κ	*disp*
Kenya	Admin. unit	-44 229 (-44 231, -44 228)	5.79 (5.56, 6.03)	1.89 (1.83, 1.96)
Kenya	20km	-43 981 (-43 984, -43 980)	25.00 (24.05, 25.97)	1.75 (1.69, 1.81)
Kenya	10km	-44 070 (-44 073, -44 069)	73.07 (70.29, 75.95)	1.80 (1.74, 1.86)
Kenya	5km	-44 193 (-44 196, -44 192)	189.43 (182.06, 197.24)	1.87 (1.81, 1.93)
Namibia	Admin. unit	-31 857 (-31 859, -31 856)	2.39 (2.31, 2.47)	2.07 (2.01, 2.14)
Namibia	20km	-34 183 (-34 186, -34 182)	20.17 (19.29, 21.08)	3.28 (3.19, 3.38)
Namibia	10km	-34 412 (-34 415, -34 411)	39.80 (38.05, 41.64)	3.43 (3.34, 3.53)
Namibia	5km	-34 654 (-34 657, -34 653)	79.23 (75.61, 83.01)	3.57 (3.47, 3.67)

Table 7. RM-v1-t0: Kenya and Namibia. Mean value and 95% credible interval (CrI) of fitted parameters of radiation model, RM-v1-t0, for Kenya and Namibia at varying spatial resolutions across all 4 MCMC chains. Parameters shown are the proportionality constant, κ, the factor of the origin population, η, and the over-dispersion parameter of the negative binomial distribution, *disp*.

Country	Scale	log-likelihood	κ	η	*disp*
Kenya	Admin. unit	-44 565 (-44 568, -44 563)	6.72 (6.64, 6.80)	0.70 (0.57, 0.86)	2.09 (2.02, 2.16)
Kenya	20km	-44 811 (-44 814, -44 809)	6.51 (6.42, 6.60)	1.08 (0.86, 1.32)	2.25 (2.18, 2.33)
Namibia	Admin. unit	-31 639 (-31 642, -31 637)	4.19 (4.16, 4.23)	3.30 (2.99, 3.61)	2.01 (1.96, 2.07)
Namibia	20km	-32 712 (-32 716, -32 711)	3.99 (3.94, 4.04)	6.51 (5.66, 7.42)	2.52 (2.45, 2.60)

Table 8. RM-v1-t1: Kenya and Namibia. Mean value and 95% credible interval (CrI) of fitted parameters of radiation model, RM-v1-t1, for Kenya and Namibia at varying spatial resolutions across all 4 MCMC chains. Parameters shown are the proportionality constant, κ, the factor of the origin population, η, and the over-dispersion parameter of the negative binomial distribution, *disp*.

Country	Scale	log-likelihood	κ	η	*disp*
Namibia	Admin. unit	-30 822 (-30 825, -30 820)	0.07 (0.05, 0.08)	17.20 (16.21, 18.22)	1.66 (1.61, 1.71)
Namibia	20km	-31 895 (-31 898, -31 894)	-0.15 (-0.18, -0.13)	23.37 (21.64, 25.15)	2.11 (2.04, 2.17)

Table 9. RM-v1-t2: Kenya and Namibia. Mean value and 95% credible interval (CrI) of fitted parameters of radiation model, RM-v1-t2, for Kenya and Namibia at varying spatial resolutions across all 4 MCMC chains. Parameters shown are the proportionality constant, κ, the power on the origin population, α, the factor of the origin population, η, and the over-dispersion parameter of the negative binomial distribution, *disp*.

Country	Scale	log-likelihood	κ	α	η	*disp*
Kenya	Admin. unit	-44 229 (-44 233, -44 227)	0.79 (0.42, 1.20)	0.99 (0.92, 1.05)	1.06 (0.89, 1.24)	1.89 (1.83, 1.96)
Kenya	20km	-44 333 (-44 337, -44 331)	-0.57 (-0.98, -0.08)	1.18 (1.10, 1.25)	1.69 (1.39, 2.01)	1.95 (1.89, 2.02)
Namibia	Admin. unit	-30 821 (-30 825, -30 819)	-0.76 (-0.98, -0.54)	1.05 (1.00, 1.10)	15.65 (14.44, 16.90)	1.65 (1.60, 1.71)
Namibia	20km	-31 999 (-32 003, -31 997)	-0.42 (-0.63, -0.20)	0.94 (0.88, 0.98)	17.78 (16.28, 19.34)	2.15 (2.09, 2.22)

Table 10. RM-v1-t3: Kenya and Namibia. Mean value and 95% credible interval (CrI) of fitted parameters of radiation model, RM-v1-t3, for Kenya and Namibia at varying spatial resolutions across all 4 MCMC chains. Parameters shown are the proportionality constant, κ, the power on the origin population, α, the factor of the origin population, η, and the over-dispersion parameter of the negative binomial distribution, *disp*.

Country	Scale	log-likelihood	κ	α	η	*disp*
Kenya	Admin. unit	-44 229 (-44 233, -44 227)	0.79 (0.42, 1.20)	0.99 (0.92, 1.05)	1.06 (0.89, 1.24)	1.89 (1.83, 1.96)
Kenya	20km	-43 865 (-43 868, -43 863)	1.87 (1.65, 2.11)	0.83 (0.79, 0.87)	2.51 (2.11, 2.92)	1.69 (1.63, 1.75)
Namibia	Admin. unit	-30 821 (-30 825, -30 819)	-0.76 (-0.98, -0.54)	1.05 (1.00, 1.10)	15.65 (14.44, 16.90)	1.65 (1.60, 1.71)
Namibia	20km	-32 095 (-32 099, -32 094)	0.95 (0.77, 1.11)	0.69 (0.65, 0.73)	44.18 (39.14, 49.76)	2.20 (2.14, 2.26)

Table 11. RM-v2-t0: Kenya and Namibia. Mean value and 95% credible interval (CrI) of fitted parameters of radiation model, RM-v2-t0, for Kenya and Namibia at varying spatial resolutions across all 4 MCMC chains. Parameters shown are the proportionality constant, κ, the increment on the origin population, θ, and the over-dispersion parameter of the negative binomial distribution, *disp*.

Country	Scale	log-likelihood	κ	θ	*disp*
Kenya	Admin. unit	-44 572 (-44 575, -44 570)	6.58 (6.56, 6.60)	1 091 (26.70, 4 080)	2.10 (2.03, 2.17)
Kenya	20km	-44 811 (-44 815, -44 810)	6.54 (6.52, 6.55)	8.27 (0.20, 31.98)	2.25 (2.18, 2.33)
Namibia	Admin. unit	-31 784 (-31 788, -31 782)	4.64 (4.63, 4.66)	49.17 (1.28, 182.57)	2.08 (2.02, 2.14)
Namibia	20km	-32 895 (-32 898, -32 893)	4.73 (4.71, 4.75)	0.57 (0.01, 2.09)	2.61 (2.54, 2.69)

Table 12. RM-v2-t1: Kenya and Namibia. Mean value and 95% credible interval (CrI) of fitted parameters of radiation model, RM-v2-t1, for Kenya and Namibia at varying spatial resolutions across all 4 MCMC chains. Parameters shown are the proportionality constant, κ, the increment on the origin population, θ, and the over-dispersion parameter of the negative binomial distribution, *disp*.

Country	Scale	log-likelihood	κ	θ	*disp*
Namibia	Admin. unit	-30 753 (-30 756, -30 751)	0.03 (0.01, 0.05)	301 361 (280 425, 323 115)	1.64 (1.59, 1.69)
Namibia	20km	-32 543 (-32 546, -32 542)	0.89 (0.87, 0.92)	288.68 (235.77, 347.09)	2.41 (2.34, 2.48)

Table 13. RM-v2-t2: Kenya and Namibia. Mean value and 95% credible interval (CrI) of fitted parameters of radiation model, RM-v2-t2, for Kenya and Namibia at varying spatial resolutions across all 4 MCMC chains. Parameters shown are the proportionality constant, κ, the power on the origin population, α, the increment on the origin population, θ, and the over-dispersion parameter of the negative binomial distribution, *disp*.

Country	Scale	log-likelihood	κ	α	θ	*disp*
Kenya	Admin. unit	-44 212 (-44 216, -44 210)	-0.55 (-1.12, 0.04)	1.20 (1.11, 1.30)	219 014 (133 558, 310 372)	1.88 (1.82, 1.95)
Kenya	20km	-44 347 (-44 351, -44 345)	-0.08 (-0.47, 0.31)	1.13 (1.07, 1.20)	14.74 (0.33, 58.59)	1.96 (1.89, 2.03)
Namibia	Admin. unit	-30 683 (-30 687, -30 682)	-1.61 (-1.81, -1.42)	1.24 (1.19, 1.28)	270 584 (251 779, 289 927)	1.61 (1.56, 1.66)
Namibia	20km	-32 573 (-32 577, -32 572)	1.82 (1.55, 2.10)	0.67 (0.61, 0.73)	11.79 (0.30, 36.54)	2.43 (2.36, 2.50)

Table 14. RM v2 t3 (called RM4 in the main text): Kenya and Namibia. Mean value and 95% credible interval (CrI) of fitted parameters of the best-fitting radiation model, RM v2 t3 (RM4 in the main text), for Kenya and Namibia at varying spatial resolutions across all 4 MCMC chains. Parameters shown are the proportionality constant, κ, the increment on the origin population, θ, the power on the origin population, α, and the over-dispersion parameter of the negative binomial distribution, *disp*.

Country	Scale	log-likelihood	κ	α	θ	*disp*
Kenya	Admin. unit	-44 212 (-44 216, -44 210)	-0.60 (-1.13, -0.03)	1.21 (1.12, 1.30)	224 628 (142 562, 311 279)	1.88 (1.82, 1.95)
Kenya	20km	-43 728 (-43 732, -43 727)	-0.88 (-1.13, -0.61)	1.34 (1.29, 1.38)	291 957 (239 482, 347 683)	1.62 (1.56, 1.67)
Kenya	10km	-43 780 (-43 783, -43 778)	-1.06 (-1.32, -0.84)	1.45 (1.41, 1.50)	252 598 (206 946, 303 149)	1.64 (1.59, 1.70)
Kenya	5km	-43 831 (-43 834, -43 829)	-1.08 (-1.28, -0.87)	1.53 (1.49, 1.57)	220 338 (176 813, 268 797)	1.67 (1.61, 1.73)
Namibia	Admin. unit	-30 683 (-30 687, -30 682)	-1.61 (-1.80, -1.43)	1.24 (1.19, 1.28)	270 557 (251 837, 289 867)	1.61 (1.56, 1.66)
Namibia	20km	-31 637 (-31 640, -31 635)	-0.80 (-0.88, -0.72)	1.10 (1.08, 1.12)	249 762 (231 136, 269 117)	2.00 (1.94, 2.06)
Namibia	10km	-31 375 (-31 379, -31 373)	-0.80 (-0.86, -0.73)	1.09 (1.07, 1.11)	287 813 (267 234, 309 234)	1.89 (1.83, 1.95)
Namibia	5km	-31 057 (-31 061, -31 055)	-0.70 (-0.75, -0.64)	1.06 (1.05, 1.08)	279 496 (259 950, 299 609)	1.76 (1.71, 1.82)

Table 15. RM v2 t4 (called RM3 in the main text): Kenya and Namibia. Mean value and 95% credible interval (CrI) of fitted parameters of radiation model, RM v2 t4 (RM3 in the main text), for Kenya and Namibia at varying spatial resolutions across all 4 MCMC chains. Parameters shown are the proportionality constant, κ, the increment on the origin population, θ, and the over-dispersion parameter of the negative binomial distribution, *disp*.

Country	Scale	log-likelihood	κ	θ	*disp*
Kenya	Admin. unit	-44 221 (-44 224, -44 220)	0.70 (0.66, 0.73)	92 451 (44 750, 145 488)	1.88 (1.82, 1.95)
Kenya	20km	-43 821 (-43 824, -43 819)	1.13 (1.09, 1.16)	66 314 (53 655, 80 549)	1.66 (1.61, 1.72)
Kenya	10km	-43 940 (-43 943, -43 938)	1.56 (1.51, 1.60)	28 039 (22 015, 34 931)	1.73 (1.67, 1.79)
Kenya	5km	-44 050 (-44 054, -44 049)	1.93 (1.88, 1.97)	13 261 (10 504, 16 439)	1.79 (1.73, 1.85)
Namibia	Admin. unit	-30 745 (-30 748, -30 743)	-0.60 (-0.61, -0.58)	273 415 (253 637, 293 690)	1.64 (1.59, 1.69)
Namibia	20km	-31 676 (-31 679, -31 675)	-0.44 (-0.46, -0.43)	248 836 (230 044, 268 537)	2.01 (1.95, 2.08)
Namibia	10km	-31 412 (-31 415, -31 410)	-0.52 (-0.54, -0.51)	289 452 (268 347, 311 163)	1.91 (1.85, 1.96)
Namibia	5km	-31 082 (-31 086, -31 081)	-0.51 (-0.53, -0.50)	283 815 (263 938, 304 426)	1.77 (1.72, 1.83)

References

[1] Simini F, González MC, Maritan A, Barabási AL. A universal model for mobility and migration patterns. Nature. 2012;484(7392):96–100. doi:10.1038/nature10856.

[2] Marshall JM, Wu SL, Sanchez C HM, Kiware SS, Ndhlovu M, Ouédraogo AL, et al. Mathematical models of human mobility of relevance to malaria transmission in Africa. Scientific Reports. 2018;8(1):7713. doi:10.1038/s41598-018-26023-1.
