# Supplementary material for: Deriving fine-scale models of human mobility from aggregated origin-destination flow data
Source: PLoS Comput Biol. 2021 Feb 11;17(2):e1008588. doi: 10.1371/journal.pcbi.1008588 (PMC7920350; doi:10.1371/journal.pcbi.1008588)
Supplement: S2 Text — Description and parameter estimates for a range of sensitivity analyses. Additional graphs for sensitivity analyses: simulated origin-destination flows and trip frequency by distance. (PDF) [file pcbi.1008588.s002.pdf]

Deriving Fine-Scale Models of Human Mobility from Aggregated Origin-Destination Flow Data
-
S2 Text: Sensitivity analyses

Constanze Ciavarella, Neil Ferguson

Contents

1 Parameter estimates for sensitivity analyses **1**

2 Simulated origin-destination flows for select sensitivity analyses **11**

3 Trip frequency by distance for select sensitivity analyses **14**

4 Flow count scatter plots for select sensitivity analyses **21**

1 Parameter estimates for sensitivity analyses

The dissimilarity between the parameter estimates of Kenya and Namibia (Fig 1 of main text) raises the question of what influences model performance. We investigate the impact of (1) population density and country geography, (2) the characteristics of empirical flow data, (3) the positioning of spatial grids. Lastly, we will look at fitting mobility models to travel data from a subset of locations. All sensitivity analyses were run on the gravity model, GM, and the best-fitting radiation model, RM-v2-t3 at the administrative unit and 20km scale.

Namibia has an unusual administrative geography and population density as compared to Kenya. The peculiarities due to its geography and travel data include (1) several small administrative units being contained within a single large administrative unit (e.g. the units making up Windhoek that are contained in a larger, doughnut-shaped unit in the Khomas Region), (2) some units being excluded due to missing travel data, (3) the salient that constitutes the region of Zambezi and part of Kavango East, and (4) heterogeneous population density. We modifying above aspects of the Namibian geography to analyse their impact on parameter estimates (Fig 1). Parameter estimates do not noticeably change if we merge administrative units that have no travel data or that have an awkward geographic form (options A, B, C and combinations thereof). However, restricting the Namibia trip dataset to administrative units in the more densely populated north causes a change in the estimates of all parameters (any scenario that contains option D). These new parameter estimates are however not closer to those computed for Kenya, which is generally more densely populated than Namibia, and the log-likelihood still worsens when moving from the administrative unit scale to the 20km scale. Comparability between the estimates of the two countries hence does not improve by restricting the geography to densely populated areas. The difference in estimates between the two countries might thus depend on the different methods by which flow counts have been extracted from CDR data by the two groups of researchers (see Materials and methods section of the main text for details).

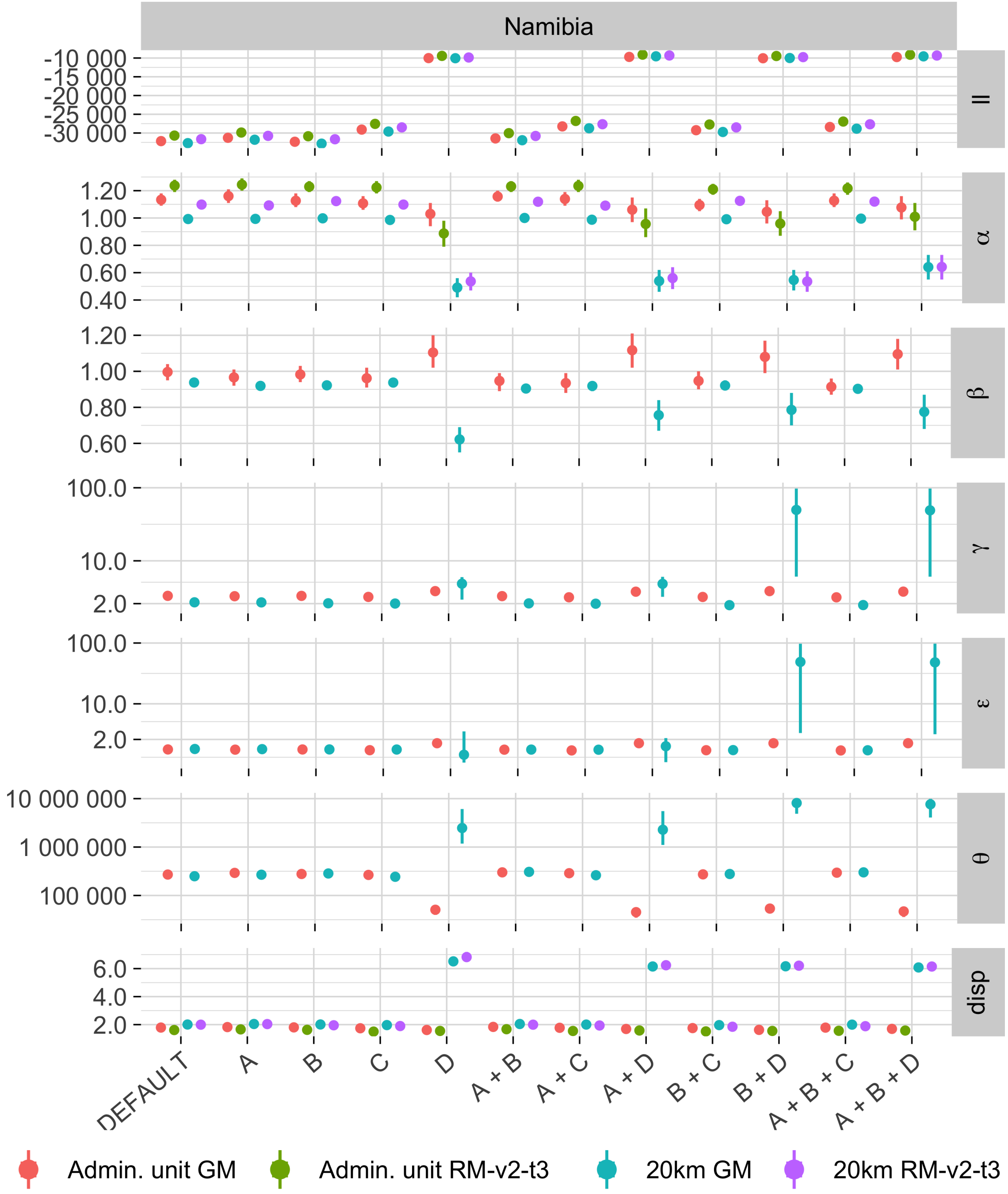

Fig 1. Alternative methods for defining the spatial geography of Namibia. (A) merge each administrative unit that is completely contained within a single other administrative unit with the containing unit; (B) merge administrative units without travel data to their closest neighbour; (C) remove administrative units in the north-eastern salient of Caprivi (contains the Zambezi region); (D) only keep administrative units in the northern regions with the highest population density. Mean value and 95% credible interval (CrI) of log-likelihood (ll) and fitted parameters of the gravity model, GM, and the best-fitting radiation model, RM-v2-t3, for Namibia at varying spatial resolutions across all 4 MCMC chains

Recreating the peculiarities of the Kenyan flow data on the Namibian data - i.e. symmetry in origin-destination flows, removing within-administrative unit trip counts, reparametrising the scaling parameter κ - causes the log-likelihood and several parameters to change substantially (Fig 2). The most far-reaching changes occur when we fit a symmetrical mobility model to symmetrised Namibian flow data (option A). Parameter *disp* decreases indicating a lower over-dispersion in the input data. For the gravity model, parameter α increases while β decreases (remember that we cannot truely distinguish between the estimates of these two parameters), thereby causing Namibian estimates to closer resemble Kenyan ones. If we look at the radiation model, there is a decrease in parameter θ, whereas parameter α increases albeit only for the administrative unit scale. Not fitting within-administrative unit trips (option B) causes the estimated value for γ to decrease for the Namibian gravity model. We notice that reparametrising the scaling factor for Kenya (option C) only causes a small shift in the estimated value for γ and ε (note that the default option for Kenya corresponds to scenario A + B + C). Applying the same shift on the gravity model for Namibia has almost no consequence.

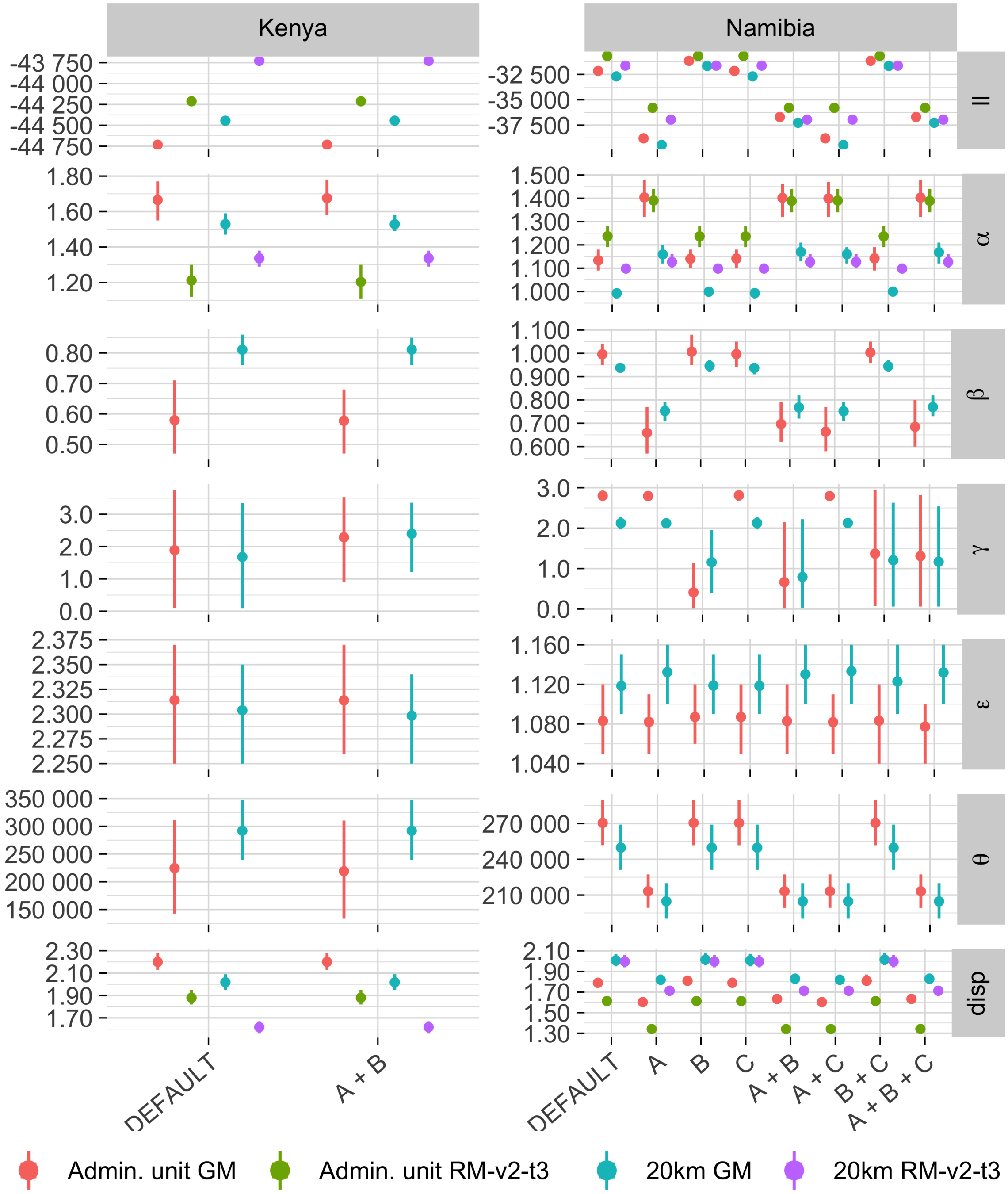

Fig 2. Modify key characteristics of the empirical input flow data. (A) symmetric data and model, i.e. sum flow data matrix with its transpose and use symmetric version of mobility model; (B) do not fit within administrative unit trips; (C) rescale the GM factor from 10^{κ} to $10^{\kappa-\gamma\varepsilon}$. N.B. for Kenya the DEFAULT corresponds to option A + B + C. Mean value and 95% credible interval (CrI) of log-likelihood (ll) and fitted parameters of the gravity model, GM, and the best-fitting radiation model, RM-v2-t3, for Kenya and Namibia at varying spatial resolutions across all 4 MCMC chains.

The choice of how to position the spatial grid can have some impact on parameter estimates and the log-likelihood (Fig 3). All 20km scale models, including the default models, are initialised from the same 4 points selected from running Latin Hypercube Sampling at the administrative unit scale, and run for 1.3 million iterations. Administrative units exist for governmental purposes and usually represent territories and communities that are highly connected. Consequently, dividing geographies using regular spatial grid risks splitting such highly connected communities. We positioned our default spatial grid in a way that was convenient for our calculations (we used the western-most and southern-most coordinates to initialise a grid), without being concerned how the capital cities or other large cities in Namibia and Kenya were being affected (Fig 4). This sensitivity analysis suggests that the choice of the positioning of the spatial grid can impact parameter estimates and log-likelihood, and should be taken into account when analysing model results at fine scales.

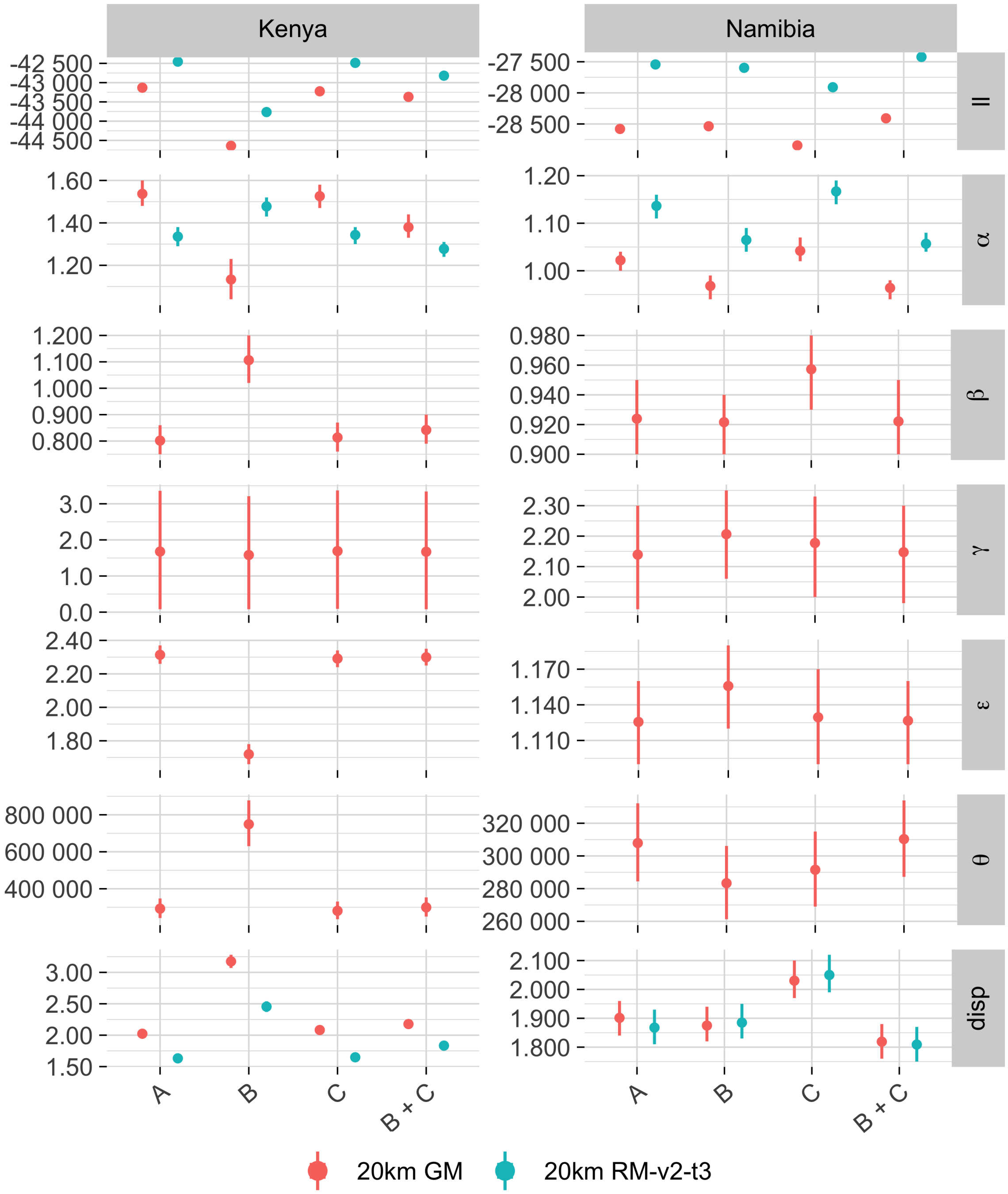

Fig 3. Modify the positioning of the 20km spatial grid. Some administrative units where not assigned any grid squares for some of the scenarios. In order to compare the different scenarios on the same data, we used the same merging method across all scenarios. (A) no shift; (B) 10 km shift in the longitude direction; (C) 10 km shift in the latitude direction. Mean value and 95% credible interval (CrI) of fitted parameters of the gravity model, GM, and the best-fitting radiation model, RM-v2-t3, for Kenya and Namibia at varying spatial resolutions across all 4 MCMC chains.

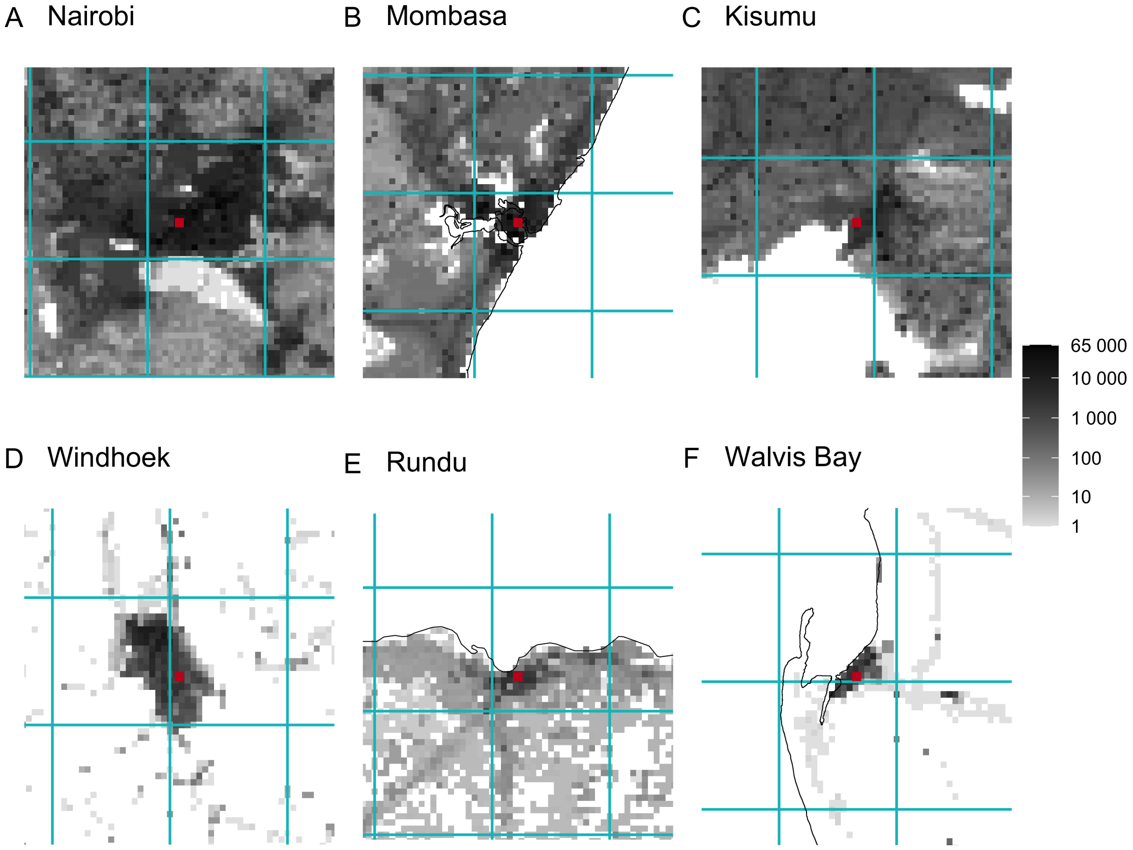

Fig 4. The positioning of the 20km spatial grid for major urban centres. Population density per $\sim 1km^2$ on a grey scale, location of the three most populous urban centres (red squares) of Kenya (top row) and Namibia (bottom row), and positioning of the default 20km grid (blue lines).

Travel data might at times be incomplete, i.e. the data might only detail trips between a number of locations. We fit the models to flow counts between a subset of randomly sampled locations from the Kenyan and Namibian dataset, and then compute the log-likelihood by applying the estimated parameters to the complete set of locations (Fig 5). Sampling 50, 30 or 15 random locations from the Kenyan and Namibian dataset, achieves satisfying fits in terms of log-likelihood. The increment on the origin population, θ, converges to very high levels for the administrative unit scales for Namibia of radiation model RM-v2-t3 for the second sampling of 15 locations (15B) for Kenya and the second sampling of 50 locations (50B). This causes an increase in the estimates of the dispersion parameter, $disp$, and a decrease in the power on the origin population, α, and a small decrease in log-likelihood. Parameter estimates for the models using 15 random locations vary somewhat for the powers on the origin and destination populations, respectively α and β. Larger sample sizes tend to provide parameter estimates closer to the ones achieved with the default model. This suggests that mobility data from a limited number of locations within a country could be sufficient to parametrise a model to estimate flows between any two given locations in that country. However, two different samples of the same size can yield somewhat different estimates for some of the parameters, as seen for the smaller sample sizes, questioning the ability of mobility models to generalise from a small dataset to a large one. Over-fitting the data is more likely to happen when the input data is limited. Hence, some of the results we see when fitting mobility model to a limited number of routes could be due to the models recreating peculiarities seen in that limited dataset.

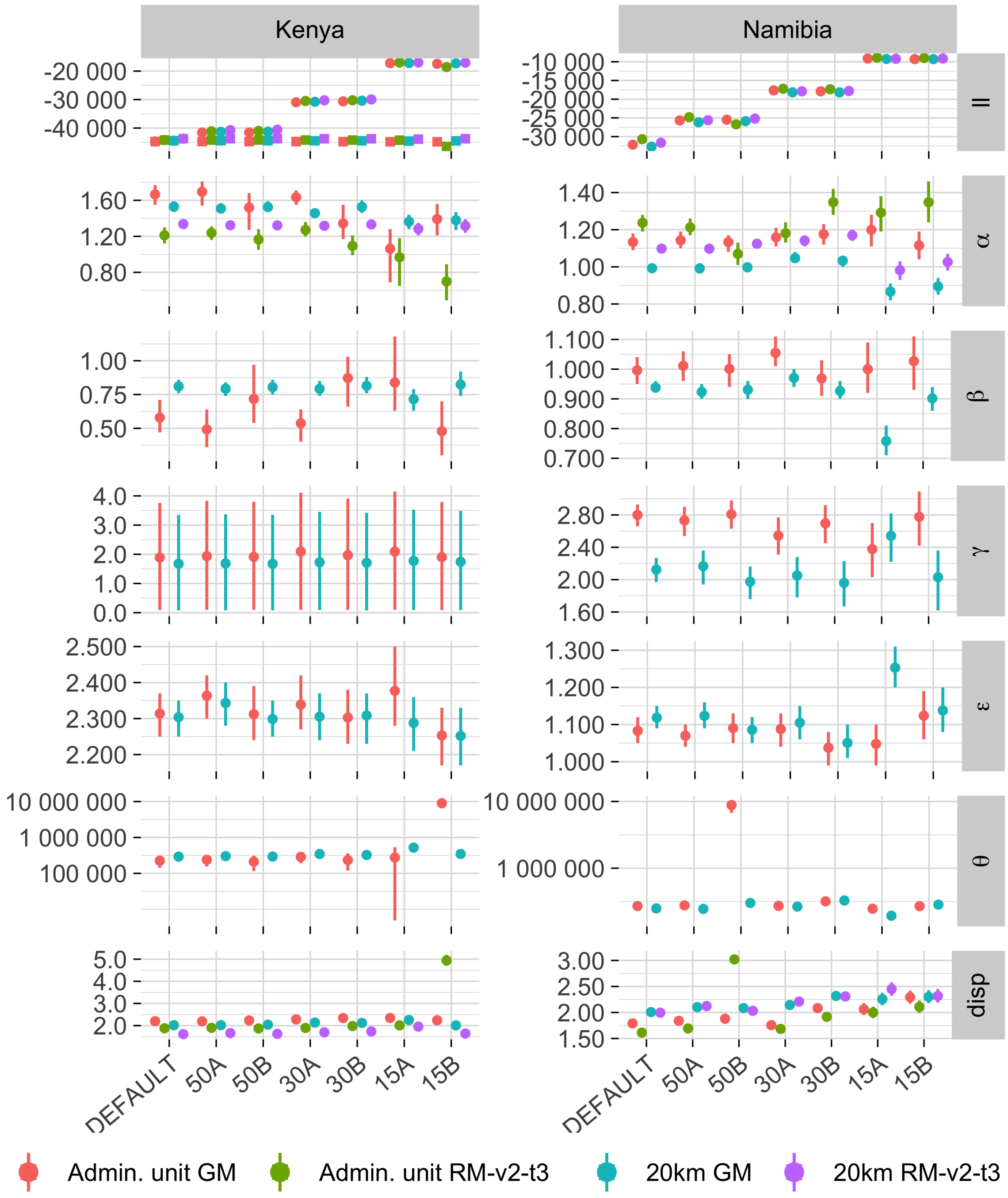

Fig 5. Fit models to flow counts between a random subset of administrative units. (50A, 50B) two separate draws of 50 administrative units, (30A, 30B) two separate draws of 30 administrative units, (15A, 15B) two separate draws of 15 administrative units. Mean value and 95% credible interval (CrI) of fitted parameters of the gravity model, GM, and the best-fitting radiation model, RM-v2-t3, for Kenya and Namibia at varying spatial resolutions across all 4 MCMC chains. Square shapes in the first row denote the log-likelihood that arises by fitting parameter estimates on a subset of the observed data and then applying these to the whole observed data.

2 Simulated origin-destination flows for select sensitivity analyses

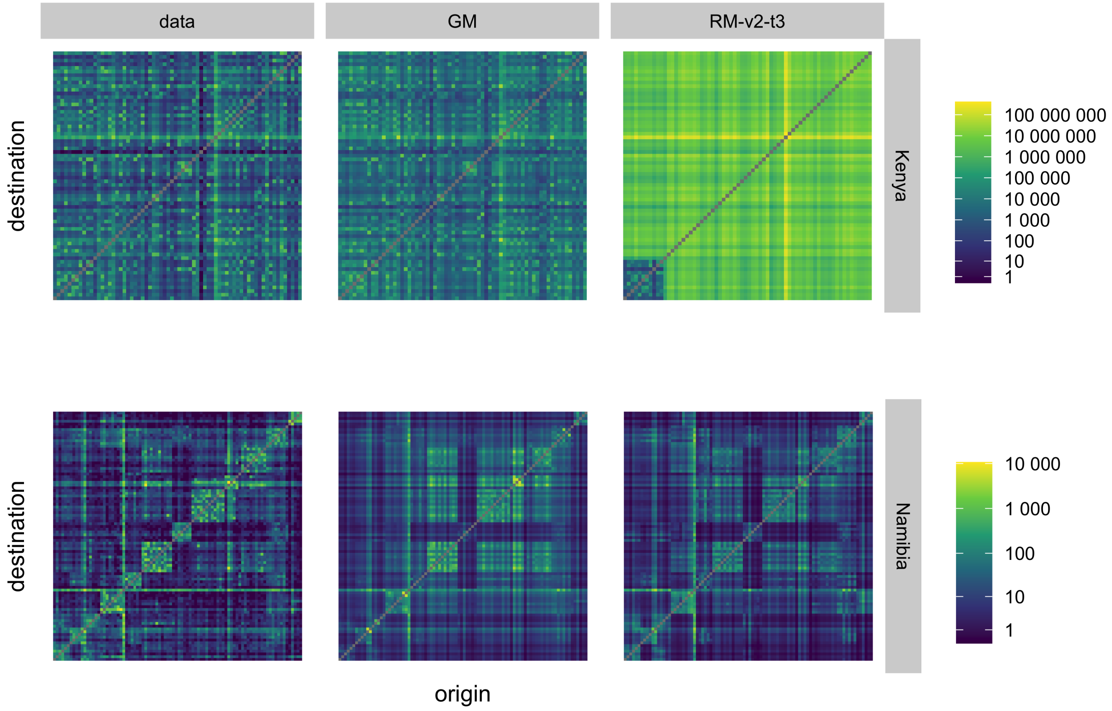

Fig 6. Simulated origin-destination flows. Origin locations are plotted along the y-axis, destination locations against the x-axis. We used administrative unit level parameter estimates to parametrise the GM and RM4 on 5km grids for Kenya and Namibia. Each pixel of the plot represents the average flow intensity across 100 parameter combination samples from the posterior distributions between the respective origin and destination administrative units. The Kenyan dataset did not report on within-unit trips, and radiation models do not predict within-unit trips; for this reason, the pixels on the diagonal, representing within-unit flows, have been removed for all models and settings. Top row: plots relative to Kenya; bottom row: plots relative to Namibia. Left column: input flow data; central column: best-fitting scale of the gravity model GM (5km scale for Kenya, administrative unit level for Namibia); right column: best-fitting scale of radiation model RM4 (20km for Kenya and administrative unit level for Namibia).

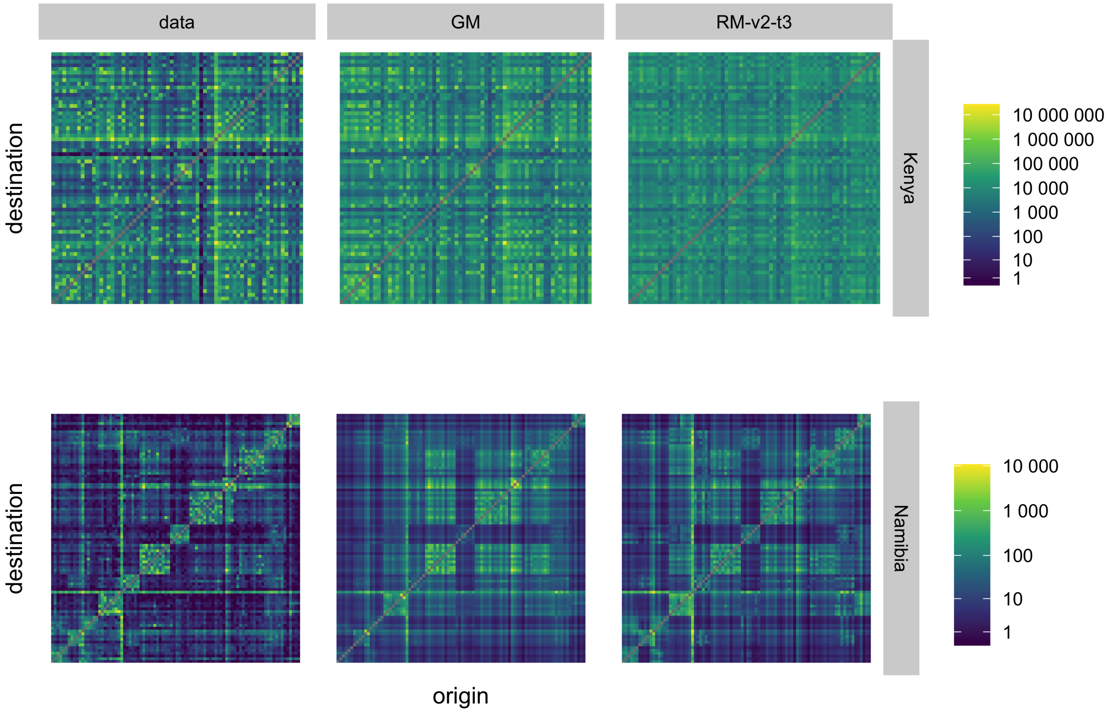

Fig 7. Simulated origin-destination flows. Origin locations are plotted along the y-axis, destination locations against the x-axis. We used parameter estimates fitted on a subset of 15 random administrative units to parametrise the GM and RM4 on all the administrative units for Kenya and Namibia. Each pixel of the plot represents the average flow intensity across 100 parameter combination samples from the posterior distributions between the respective origin and destination administrative units. The Kenyan dataset did not report on within-unit trips, and radiation models do not predict within-unit trips; for this reason, the pixels on the diagonal, representing within-unit flows, have been removed for all models and settings. Top row: plots relative to Kenya; bottom row: plots relative to Namibia. Left column: input flow data; central column: best-fitting scale of the gravity model GM (5km scale for Kenya, administrative unit level for Namibia); right column: best-fitting scale of radiation model RM4 (20km for Kenya and administrative unit level for Namibia).

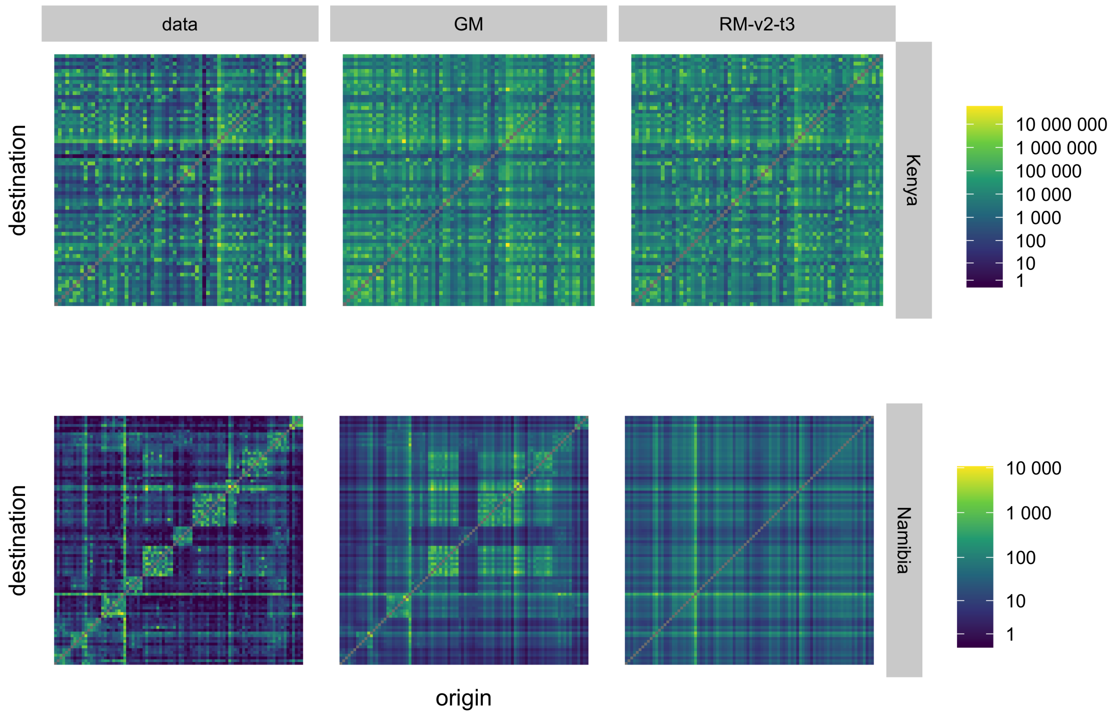

Fig 8. Simulated origin-destination flows. Origin locations are plotted along the y-axis, destination locations against the x-axis. We used parameter estimates fitted on a subset of 50 random administrative units to parametrise the GM and RM4 on all the administrative units for Kenya and Namibia. Each pixel of the plot represents the average flow intensity across 100 parameter combination samples from the posterior distributions between the respective origin and destination administrative units. The Kenyan dataset did not report on within-unit trips, and radiation models do not predict within-unit trips; for this reason, the pixels on the diagonal, representing within-unit flows, have been removed for all models and settings. Top row: plots relative to Kenya; bottom row: plots relative to Namibia. Left column: input flow data; central column: best-fitting scale of the gravity model GM (5km scale for Kenya, administrative unit level for Namibia); right column: best-fitting scale of radiation model RM4 (20km for Kenya and administrative unit level for Namibia).

3 Trip frequency by distance for select sensitivity analyses

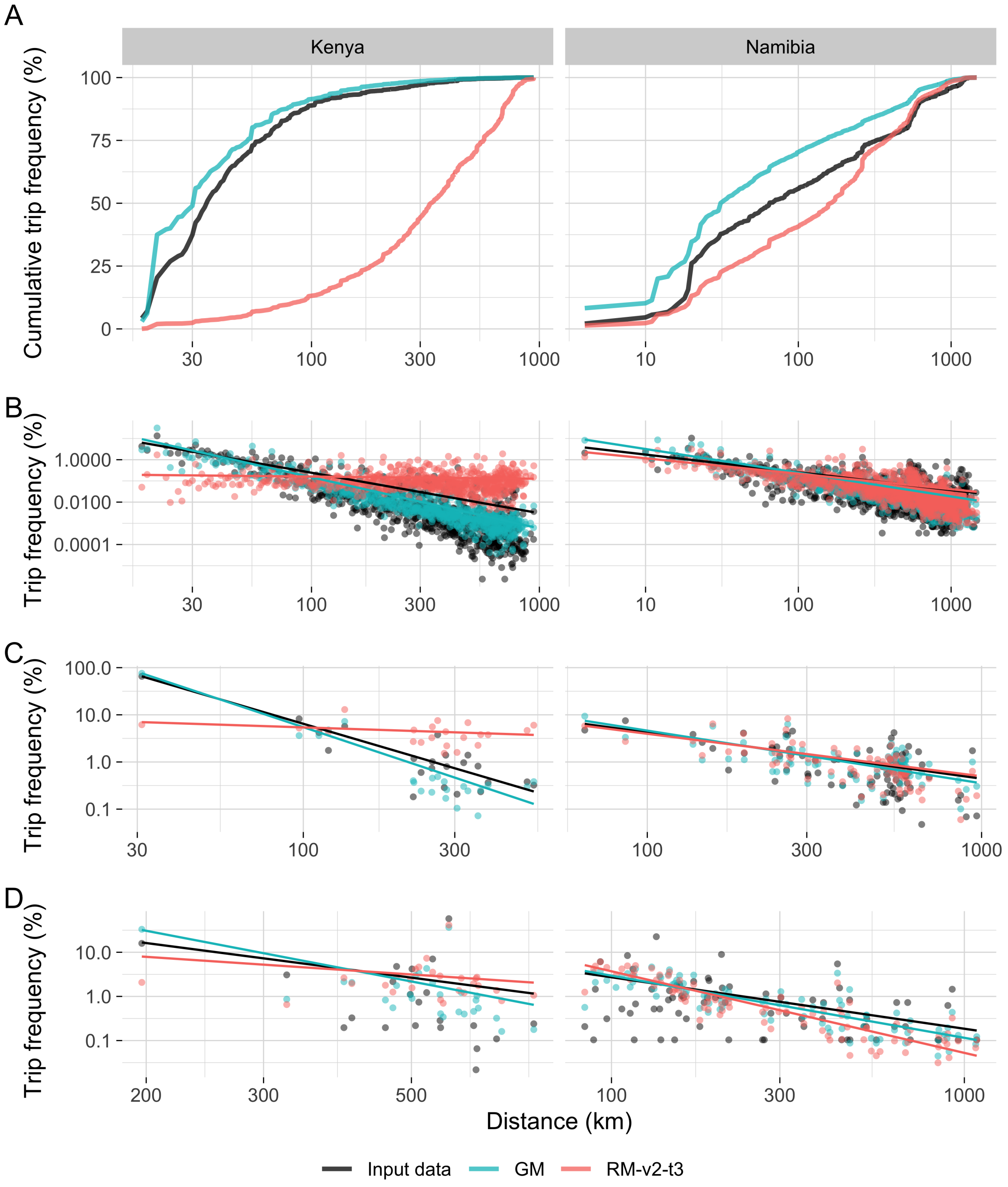

Fig 9. Trip frequency by distance. Empirical data (black) and simulated data using the gravity model GM (blue) and radiation model RM4 (red). We used administrative unit level parameter estimates to parametrise the GM and RM4 on 5km grids for Kenya and Namibia. Left column panels relate to Kenya, right column panels to Namibia. (A) Cumulative trip frequency by distance across all origin-destination pairs. (B) Trip frequency by distance across all origin-destination pairs. (C) Trip frequency by distance restricted to trips originating in the administrative unit where the most trips start (Nairobi Province (now Nairobi County) for Kenya, the union of Windhoek rural and Windhoek west constituencies of the Khomas region for Namibia). (D) Trip frequency by distance restricted to trips originating in the administrative unit where the fewest trips start (Moyale District (now part of Marsabit County) for Kenya, Uuvudhiya Constituency of the Oshana region for Namibia).

Table 1. Estimated parameter values of the power law fit in Fig 9.

Panel	Country	Model	Exponent
B	Kenya	data	-1.91
B	Kenya	GM	-2.41
B	Kenya	RM-v2-t3	-0.11
B	Namibia	data	-0.84
B	Namibia	GM	-1.12
B	Namibia	RM-v2-t3	-0.73
C	Kenya	data	-1.98
C	Kenya	GM	-2.24
C	Kenya	RM-v2-t3	-0.22
C	Namibia	data	-0.98
C	Namibia	GM	-1.12
C	Namibia	RM-v2-t3	-0.90
D	Kenya	data	-1.97
D	Kenya	GM	-2.89
D	Kenya	RM-v2-t3	-1.00
D	Namibia	data	-1.17
D	Namibia	GM	-1.41
D	Namibia	RM-v2-t3	-1.85

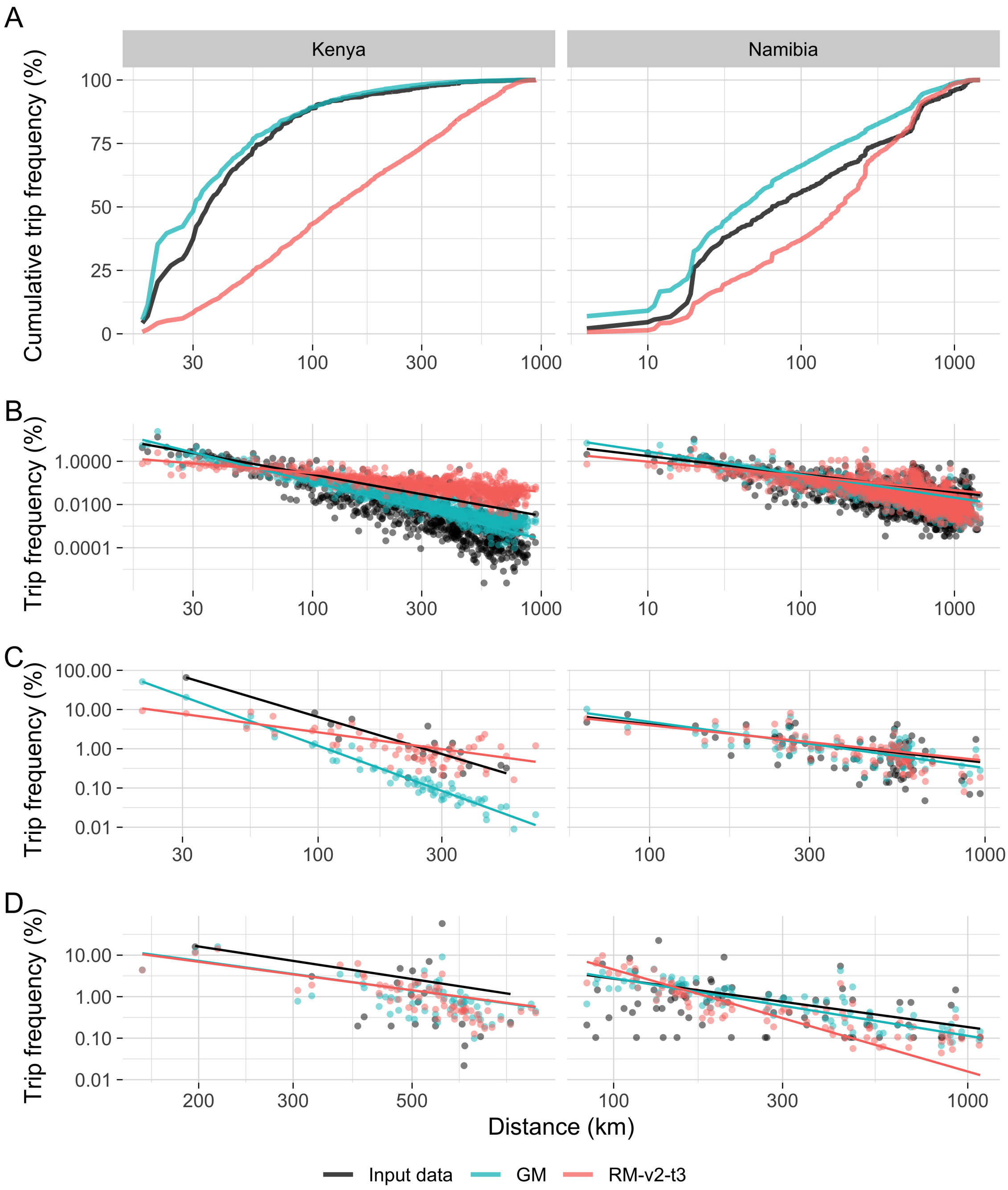

Fig 10. Trip frequency by distance. Empirical data (black) and simulated data using the gravity model GM (blue) and radiation model RM4 (red). We used parameter estimates fitted on a subset of 15 random administrative units to parametrise the GM and RM4 on all the administrative units for Kenya and Namibia. Left column panels relate to Kenya, right column panels to Namibia. (A) Cumulative trip frequency by distance across all origin-destination pairs. (B) Trip frequency by distance across all origin-destination pairs. (C) Trip frequency by distance restricted to trips originating in the administrative unit where the most trips start (Nairobi Province (now Nairobi County) for Kenya, the union of Windhoek rural and Windhoek west constituencies of the Khomas region for Namibia). (D) Trip frequency by distance restricted to trips originating in the administrative unit where the fewest trips start (Moyale District (now part of Marsabit County) for Kenya, Uuvudhiya Constituency of the Oshana region for Namibia).

Table 2. Estimated parameter values of the power law fit in Fig 10.

Panel	Country	Model	Exponent
B	Kenya	data	-1.91
B	Kenya	GM	-2.63
B	Kenya	RM-v2-t3	-0.86
B	Namibia	data	-0.84
B	Namibia	GM	-1.07
B	Namibia	RM-v2-t3	-0.66
C	Kenya	data	-1.98
C	Kenya	GM	-2.41
C	Kenya	RM-v2-t3	-0.90
C	Namibia	data	-0.98
C	Namibia	GM	-1.18
C	Namibia	RM-v2-t3	-0.90
D	Kenya	data	-1.97
D	Kenya	GM	-1.78
D	Kenya	RM-v2-t3	-1.72
D	Namibia	data	-1.17
D	Namibia	GM	-1.39
D	Namibia	RM-v2-t3	-2.46

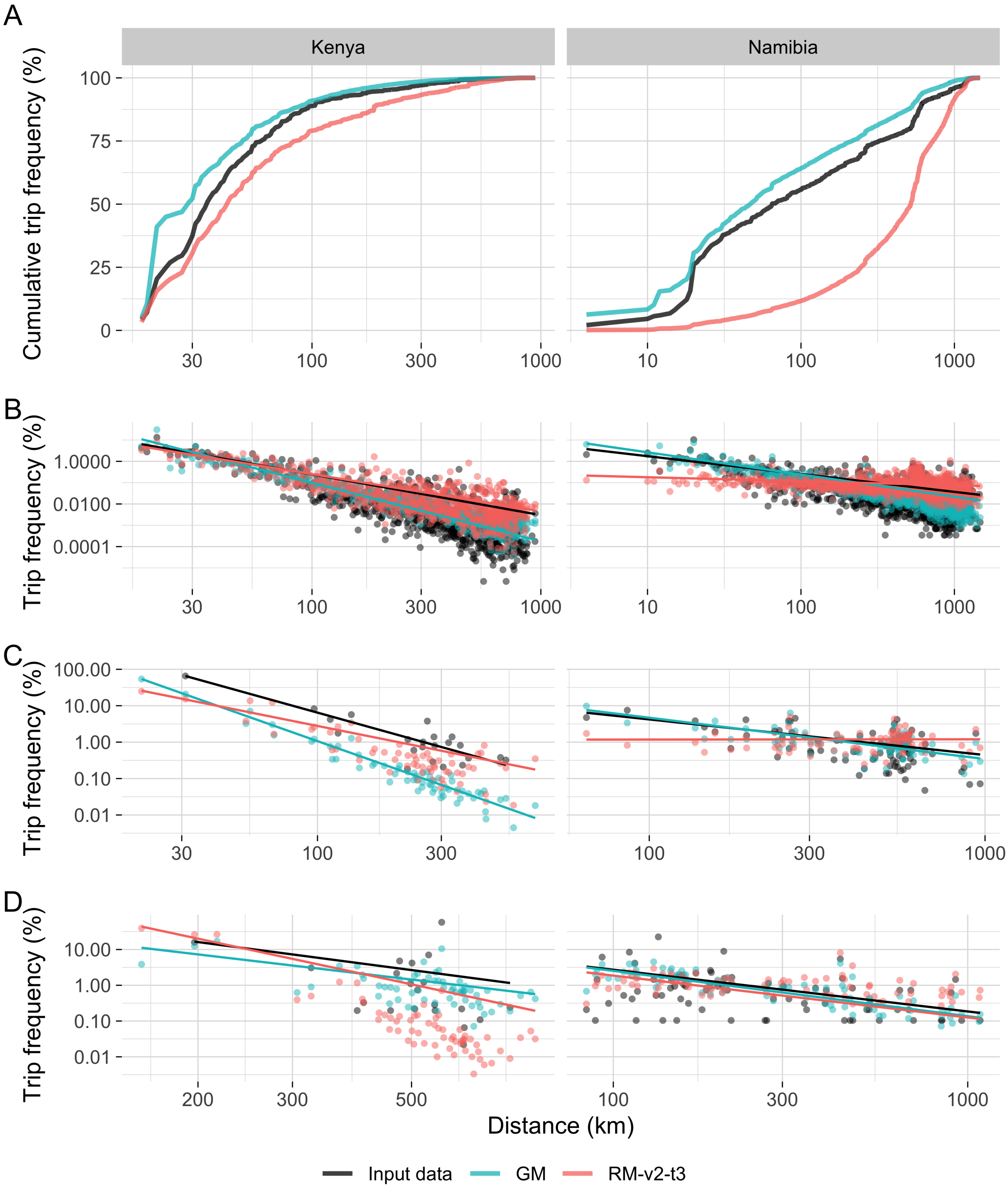

Fig 11. Trip frequency by distance. Empirical data (black) and simulated data using the gravity model GM (blue) and radiation model RM4 (red). We used parameter estimates fitted on a subset of 50 random administrative units to parametrise the GM and RM4 on all the administrative units for Kenya and Namibia. Left column panels relate to Kenya, right column panels to Namibia. (A) Cumulative trip frequency by distance across all origin-destination pairs. (B) Trip frequency by distance across all origin-destination pairs. (C) Trip frequency by distance restricted to trips originating in the administrative unit where the most trips start (Nairobi Province (now Nairobi County) for Kenya, the union of Windhoek rural and Windhoek west constituencies of the Khomas region for Namibia). (D) Trip frequency by distance restricted to trips originating in the administrative unit where the fewest trips start (Moyale District (now part of Marsabit County) for Kenya, Uuvudhiya Constituency of the Oshana region for Namibia).

Table 3. Estimated parameter values of the power law fit in Fig 11.

Panel	Country	Model	Exponent
B	Kenya	data	-1.91
B	Kenya	GM	-2.75
B	Kenya	RM-v2-t3	-1.74
B	Namibia	data	-0.84
B	Namibia	GM	-1.04
B	Namibia	RM-v2-t3	-0.19
C	Kenya	data	-1.98
C	Kenya	GM	-2.52
C	Kenya	RM-v2-t3	-1.43
C	Namibia	data	-0.98
C	Namibia	GM	-1.13
C	Namibia	RM-v2-t3	0.01
D	Kenya	data	-1.97
D	Kenya	GM	-1.77
D	Kenya	RM-v2-t3	-3.21
D	Namibia	data	-1.17
D	Namibia	GM	-1.27
D	Namibia	RM-v2-t3	-1.17

4 Flow count scatter plots for select sensitivity analyses

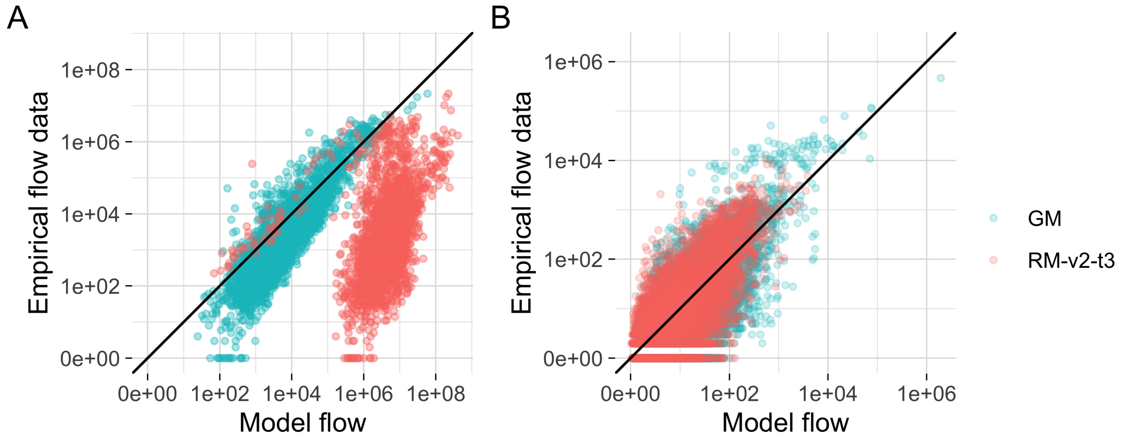

Fig 12. Empirical vs. modelled flow counts at 5km scale. We used administrative unit level parameter estimates to parametrise the GM and RM-v2-t3 on 5km grids for Kenya and Namibia. Modelled flow counts are computed as the mean across the flows resulting from 100 parameter combinations sampled from the posterior distributions of the models. The Kenyan dataset did not report on within-unit trips, and radiation models do not predict within-unit trips. (A) Kenya, (B) Namibia. Note that since the mobility models for Kenya are symmetric, we plot each point twice and therefore they appear to be darker in panel A than in panel B.

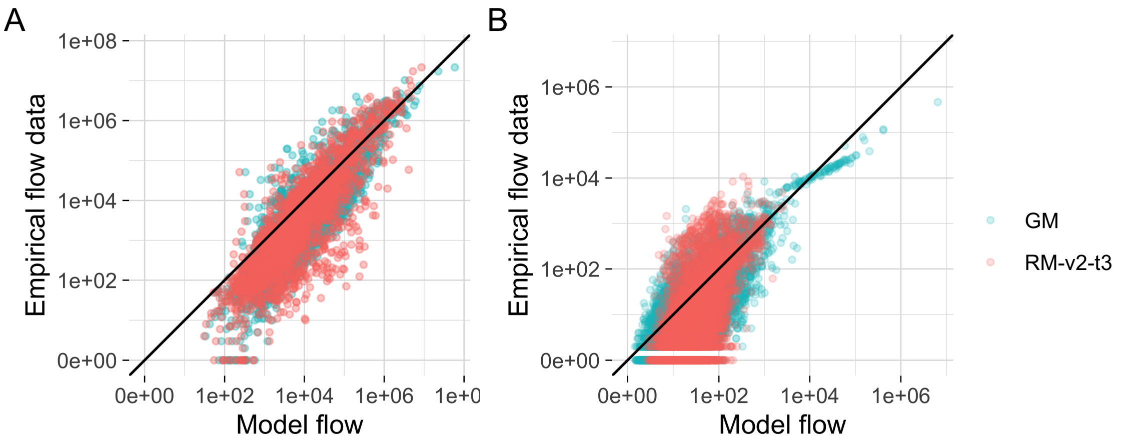

Fig 13. Empirical vs. modelled flow counts fitted on 50 random administrative units. We used parameter estimates fitted on a subset of 50 random administrative units to parametrise the GM and RM-v2-t3 on all the administrative units for Kenya and Namibia. Modelled flow counts are computed as the mean across the flows resulting from 100 parameter combinations sampled from the posterior distributions of the models. The Kenyan dataset did not report on within-unit trips, and radiation models do not predict within-unit trips. (A) Kenya, (B) Namibia. Note that since the mobility models for Kenya are symmetric, we plot each point twice and therefore they appear to be darker in panel A than in panel B.

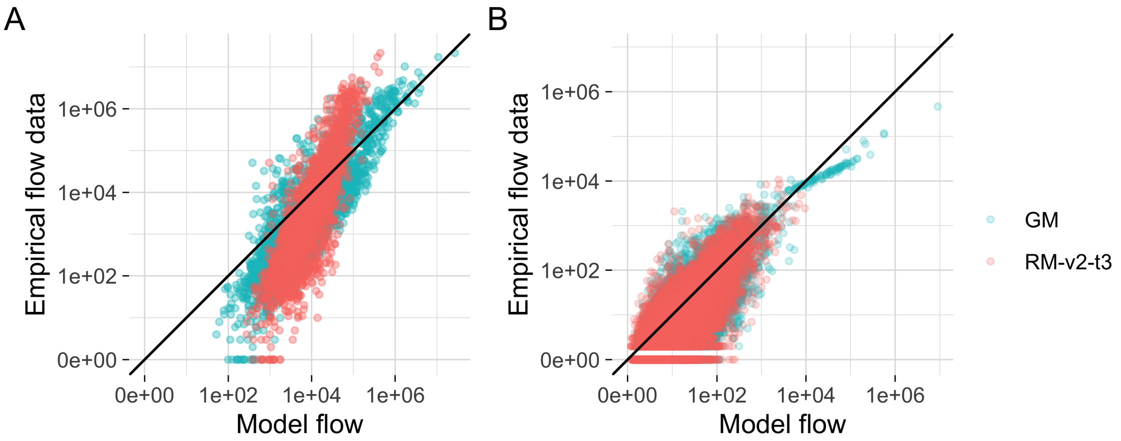

Fig 14. Empirical vs. modelled flow counts fitted on 15 random administrative units. We used parameter estimates fitted on a subset of 15 random administrative units to parametrise the GM and RM-v2-t3 on all the administrative units for Kenya and Namibia. Modelled flow counts are computed as the mean across the flows resulting from 100 parameter combinations sampled from the posterior distributions of the models. The Kenyan dataset did not report on within-unit trips, and radiation models do not predict within-unit trips. (A) Kenya, (B) Namibia. Note that since the mobility models for Kenya are symmetric, we plot each point twice and therefore they appear to be darker in panel A than in panel B.
