# Supplementary material for: Deriving fine-scale models of human mobility from aggregated origin-destination flow data
Source: PLoS Comput Biol. 2021 Feb 11;17(2):e1008588. doi: 10.1371/journal.pcbi.1008588 (PMC7920350; doi:10.1371/journal.pcbi.1008588)
Supplement: S1 Table — (PDF) [file pcbi.1008588.s003.pdf]

S1 Table. Proportion of origin-destination flow counts that fall within the 95% credible intervals (CrIs) of the estimated flows.

Country	Model	Scale	Lower than 95% CrI (%)	Within 95% CrI (%)	Higher than 95% CrI (%)
Kenya	GM	10km	78.20	6.23	15.57
Kenya	RM-v2-t3	20km	74.22	5.97	19.81
Namibia	GM	Admin. unit	71.83	5.95	22.22
Kenya	GM	5km	77.68	5.88	16.44
Kenya	GM	20km	78.37	5.84	15.79
Kenya	RM-v2-t3	10km	76.04	5.80	18.17
Kenya	GM	Admin. unit	80.06	5.41	14.53
Namibia	GM	5km	74.00	5.26	20.74
Namibia	GM	10km	73.48	5.14	21.38
Namibia	RM-v2-t3	Admin. unit	69.51	5.00	25.49
Namibia	GM	20km	73.81	4.89	21.30
Kenya	RM-v2-t3	Admin. unit	75.56	4.84	19.59
Namibia	RM-v2-t3	5km	71.00	4.19	24.81
Namibia	RM-v2-t3	20km	74.05	4.15	21.80
Namibia	RM-v2-t3	10km	73.14	3.90	22.96
Kenya	RM-v2-t3	5km	98.10	1.82	0.09
