# Supplementary material for: Deriving fine-scale models of human mobility from aggregated origin-destination flow data
Source: PLoS Comput Biol. 2021 Feb 11;17(2):e1008588. doi: 10.1371/journal.pcbi.1008588 (PMC7920350; doi:10.1371/journal.pcbi.1008588)
Supplement: S2 Table — (PDF) [file pcbi.1008588.s004.pdf]

S2 Table. Estimated parameter values of the power law fit in Fig 3 of the main text.

Panel	Country	Model	Exponent
A	Kenya	data	-1.91
A	Kenya	GM	-2.54
A	Kenya	RM-v2-t3	-1.94
A	Namibia	data	-0.84
A	Namibia	GM	-1.03
A	Namibia	RM-v2-t3	-0.67
B	Kenya	data	-1.98
B	Kenya	GM	-2.46
B	Kenya	RM-v2-t3	-1.09
B	Namibia	data	-0.98
B	Namibia	GM	-1.12
B	Namibia	RM-v2-t3	-0.90
C	Kenya	data	-1.97
C	Kenya	GM	-2.78
C	Kenya	RM-v2-t3	-4.64
C	Namibia	data	-1.17
C	Namibia	GM	-1.19
C	Namibia	RM-v2-t3	-2.43
