# Supplementary material for: Deriving fine-scale models of human mobility from aggregated origin-destination flow data
Source: PLoS Comput Biol. 2021 Feb 11;17(2):e1008588. doi: 10.1371/journal.pcbi.1008588 (PMC7920350; doi:10.1371/journal.pcbi.1008588)
Supplement: S1 Fig — Top row: cumulative frequency of cells by their population; bottom row: cumulative proportion of population contained in cells with a log population size smaller or equal than the values reported on the x-axis. Columns refer to different spatial scales: administrative unit, 20km, 10km and 5km scales. Data for Kenya is in orange, data for Namibia in purple. (PDF) [file pcbi.1008588.s005.pdf]

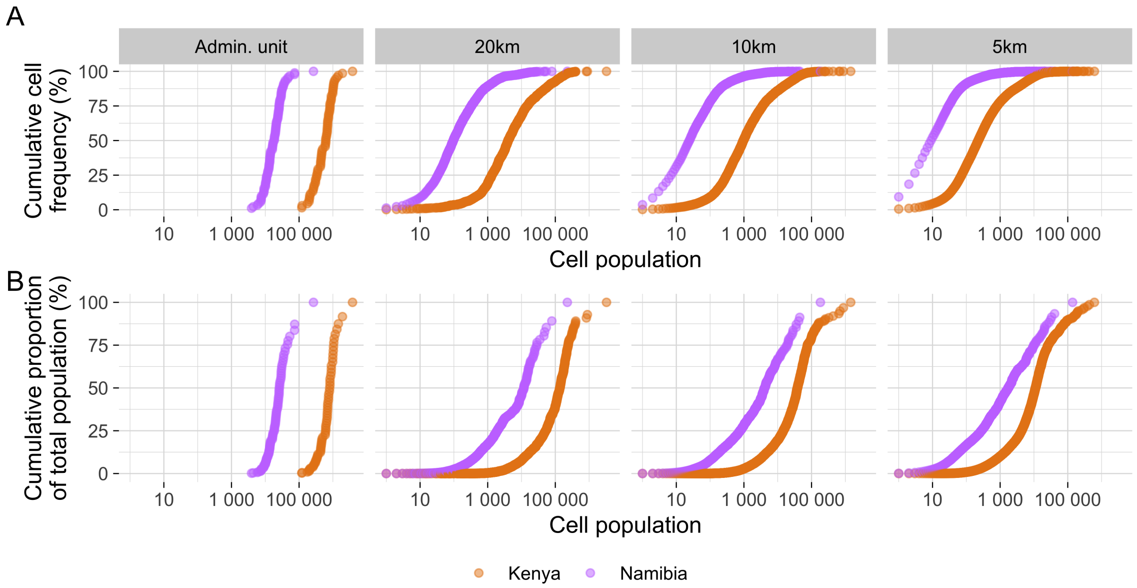

S1 Fig. Distribution of cell population across spatial scales. Top row: cumulative frequency of cells by their population; bottom row: cumulative proportion of population contained in cells with a log population size smaller or equal than the values reported on the x-axis. Columns refer to different spatial scales: administrative unit, 20km, 10km and 5km scales. Data for Kenya is in orange, data for Namibia in purple.
