# Supplementary material for: Deriving fine-scale models of human mobility from aggregated origin-destination flow data
Source: PLoS Comput Biol. 2021 Feb 11;17(2):e1008588. doi: 10.1371/journal.pcbi.1008588 (PMC7920350; doi:10.1371/journal.pcbi.1008588)
Supplement: S2 Fig — We plotted the best-fitting scale of the gravity model, GM, (5km scale for Kenya, administrative unit level for Namibia) and the best-fitting scale of radiation model, RM4, (20km for Kenya and administrative unit level for Namibia). Modelled flow counts are computed as the mean across the flows resulting from 100 parameter combinations sampled from the posterior distributions of the models. The Kenyan dataset did not report on within-unit trips, and radiation models do not predict within-unit trips. (A) Kenya, (B) Namibia. Note that since the mobility models for Kenya are symmetric, we plot each point twice and therefore they appear to be darker in panel A than in panel B. (PDF) [file pcbi.1008588.s006.pdf]

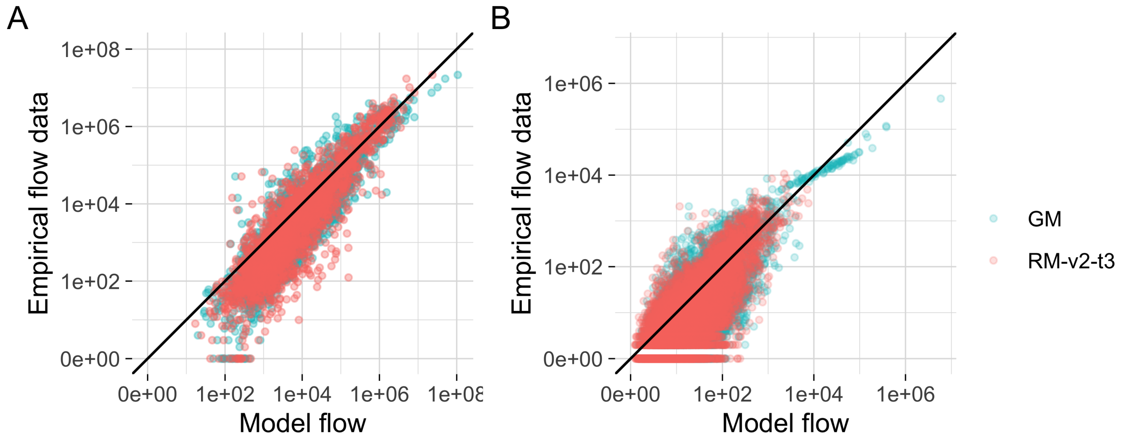

S2 Fig. Empirical vs. modelled flow counts. We plotted the best-fitting scale of the gravity model GM (5km scale for Kenya, administrative unit level for Namibia) and the best-fitting scale of radiation model RM-v2-t3 (20km for Kenya and administrative unit level for Namibia). Modelled flow counts are computed as the mean across the flows resulting from 100 parameter combinations sampled from the posterior distributions of the models. The Kenyan dataset did not report on within-unit trips, and radiation models do not predict within-unit trips. (A) Kenya, (B) Namibia. Note that since the mobility models for Kenya are symmetric, we plot each point twice and therefore they appear to be darker in panel A than in panel B.
