# Supplementary material for: Deriving fine-scale models of human mobility from aggregated origin-destination flow data
Source: PLoS Comput Biol. 2021 Feb 11;17(2):e1008588. doi: 10.1371/journal.pcbi.1008588 (PMC7920350; doi:10.1371/journal.pcbi.1008588)
Supplement: S3 Fig — Each pixel represent an origin-destination administrative unit pair. Origin locations are plotted along the x-axis, destination locations against the y-axis. Let i and j be an origin and a destination location, and let fij be the flow from i to j as reported in the empirical data. For each i and j we compute Fij: = (fij − fji)/max(fij, fji) and plot it. This yields a value between -100 and 100: red and orange pixels (values > 25) indicate that the journeys from i to j where considerably more numerous that the journeys from j to i, dark blue and bluish pixels (values < -25) indicate that the journeys from j to i where considerably more numerous that the journeys from i to j, yellow pixels (-25 < values < 25) indicate that the journeys form i to j where about as numerous as the journeys from j to i demonstrating thus that the flow between the two administrative units is symmetric. For Kenya the absolute value of values Fij is 11.04 (95%CrI 0.00-42.86), whereas for Namibia the average lies at 45.59 (95%CrI 0.00-100.00). (PDF) [file pcbi.1008588.s007.pdf]

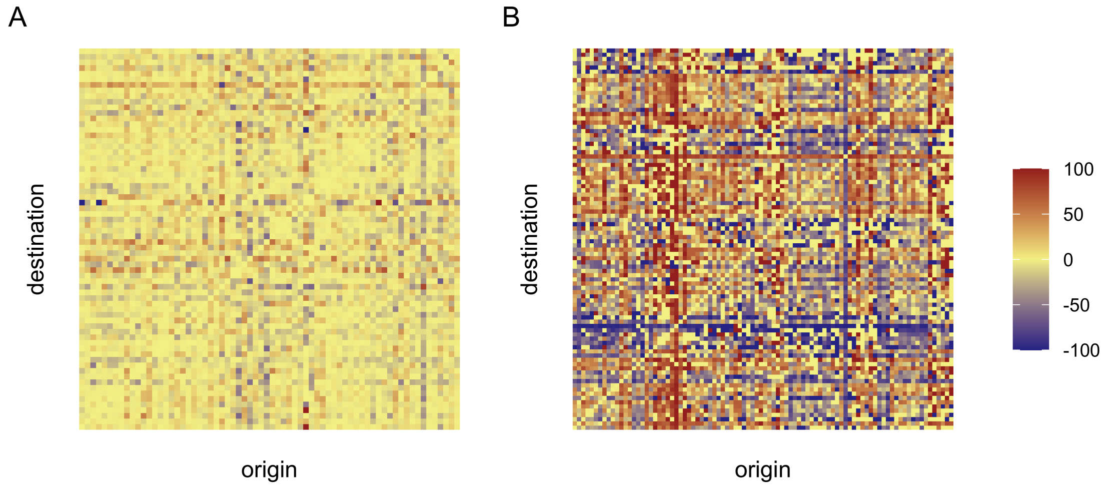

S3 Fig. Symmetry of the origin-destination matrices for Kenya (left) and Namibia (right). Each pixel represent an origin-destination administrative unit pair. Origin locations are plotted along the y-axis, destination locations against the x-axis. Let i and j be an origin and a destination location, and let f_{ij} be the flow from i to j as reported in the empirical data. For each i and j we compute $F_{ij} := (f_{ij} - f_{ji})/max(f_{ij}, f_{ji})$ and plot it. This yields a value between -100 and 100: red and orange pixels (values $>$ 25) indicate that the journeys from i to j where considerably more numerous that the journeys from j to i, dark blue and bluish pixels (values $<$ -25) indicate that the journeys from j to i where considerably more numerous that the journeys from i to j, yellow pixels (-25 $<$ values $<$ 25) indicate that the journeys form i to j where about as numerous as the journeys from j to i demonstrating thus that the flow between the two administrative units is symmetric. For Kenya the absolute value of values F_{ij} is 11.04 (95%CrI 0.00-42.86), whereas for Namibia the average lies at 45.59 (95%CrI 0.00-100.00).
