# Supplementary material for: Deriving fine-scale models of human mobility from aggregated origin-destination flow data
Source: PLoS Comput Biol. 2021 Feb 11;17(2):e1008588. doi: 10.1371/journal.pcbi.1008588 (PMC7920350; doi:10.1371/journal.pcbi.1008588)
Supplement: S4 Fig — Left column: default model fitted for Kenya (as described in the main text); right column: we implemented the same model with the exception that the model scaling factor was 10κ. The 10km and 5km grid scales were not fit for the model in the right column. (PDF) [file pcbi.1008588.s008.pdf]

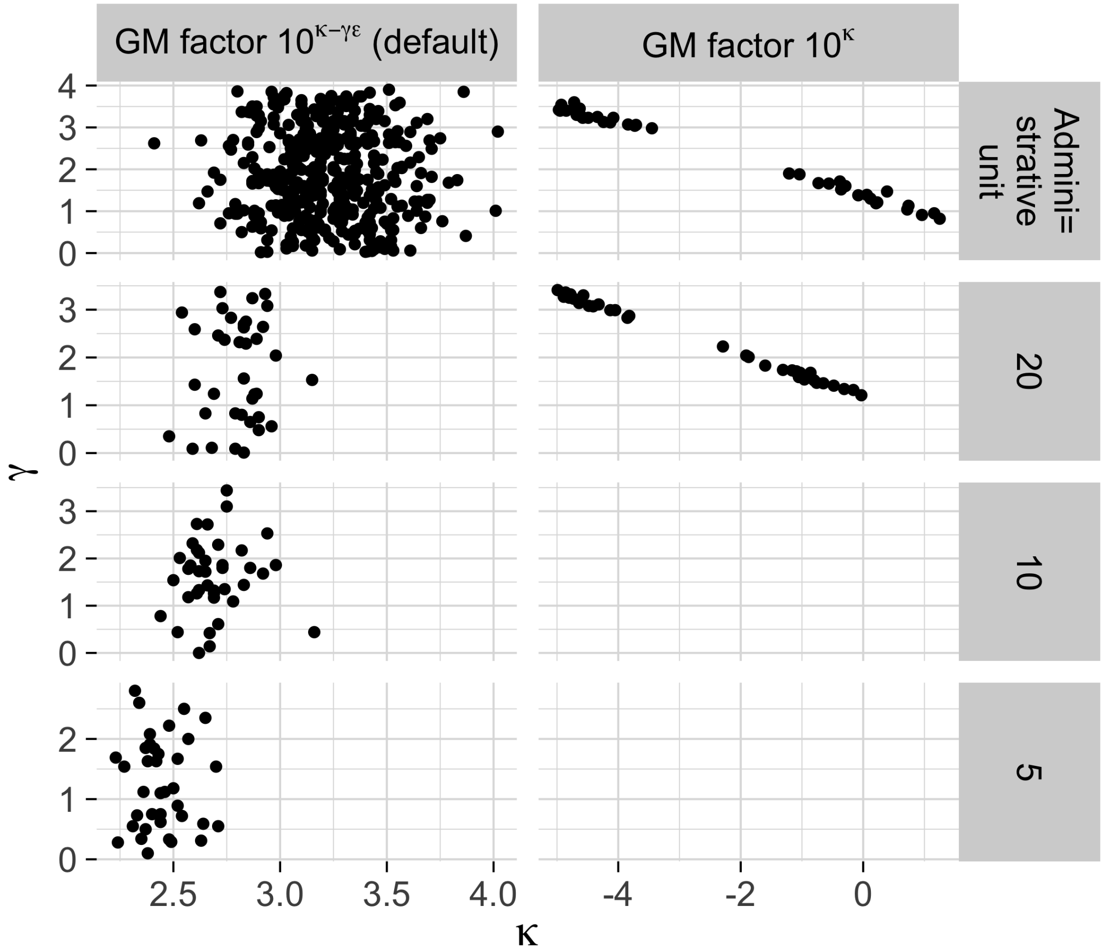

S4 Fig. Trade-off between parameters κ and γ for the gravity model, GM, for Kenya. Left column: default model fitted for Kenya (as described in the main text); right column: we implemented the same model with the exception that the model scaling factor was 10^{κ}. The 10km and 5km grid scales were not fit for the model in the right column.
