# Supplementary material for: Deriving fine-scale models of human mobility from aggregated origin-destination flow data
Source: PLoS Comput Biol. 2021 Feb 11;17(2):e1008588. doi: 10.1371/journal.pcbi.1008588 (PMC7920350; doi:10.1371/journal.pcbi.1008588)
Supplement: Attachment — Submitted filename: Response to Reviewers.docx [file pcbi.1008588.s009.docx]

# Response to Reviewers

## Deriving fine-scale models of human mobility form aggregated origin-destination flow data

### Constanze Ciavarella, Neil M Ferguson

We thank both reviewers for their comments and suggestions. We improved how results are presented in the text and figures of the main file. A great number of sensitivity analyses have been carried out. These results are presented in the supplementary files and cited in the main text where appropriate.

We hope to have answered the reviewer's questions and concerns adequately.

## Reviewer #1:

**In “Deriving fine-scale models of human mobility from aggregated origin-destination flow data”, the authors use aggregated mobility data to develop a finer spatial scale model of mobility. The authors build off of the two most commonly applied spatial interaction models (gravity model and radiation model) and use those frameworks to estimate travel at a finer spatial scale. The authors use population and distance data to estimate these travel patterns (down to 1 km). Using previously published mobility data calculated using mobile phone calling records, the authors first fit spatial interaction models to the origin-destination trip counts. The authors the use these origin-destination trip counts to assume that the sum of travel on finer spatial scales is equal to the aggregate. By assuming the finer scale data is equal to the sum of the aggregate, they then fit the same spatial interaction models to these data. Overall, the analysis presented is adequate but would be greatly strengthened by 1) elaborating on the implications and interpretation of the models fit, 2) providing quantitative results of the implications of these finer scale models, and 3) demonstrating the implications for a biological purpose. The primary concern for the methods is the omission (not the fault of the authors) of within administrative unit travel and the implications on the parameters estimated.**

Major Comments:

**a) The authors do not analyze any travel within a spatial unit. Although the authors state that the within administrative unit data is unavailable (based on the published work where the authors obtained these data), this omission (which is not the fault of the authors) may be biasing estimates from the models. In particular, the role of distance and the estimated parameter (since very short distances – or distances of length 0) may be impacted. On the larger spatial scale of the data, this omission may be less relevant since travel is likely to occur over larger distances (given the administrative unit geography). However, this assumption (that within spatial unit travel can be omitted from the analysis), may be less valid for the finer spatial units analyzed. For example, on the 1km grid scale, very local travel is highly likely since these represent very close and small spatial units. Including data on these scales would likely down weight the distance parameter. However, travel patterns on these scales are not included since they were not included in the originally published data set. Hence the authors cannot state that they are estimating travel between these fine spatial units when actually they are estimating travel between fine spatial units assuming that travel only occurs between large spatial units. This fact underpins the entire analysis, yet the authors do not carefully and clearly address these points in the analysis.**

**We thank the reviewer for this comment that highlights some of the weaknesses of the analysis and the communication of its results. We added the below paragraph to the discussion on lines 201-208:**

Radiation models do not estimate within-unit trips, and the Kenyan dataset does not provide data on within-unit travel. Hence, we were only able to fit the gravity model to Namibian within-unit trip counts. This also applies to trips between cells belonging to the same unit at the grid scales.

Recreating the peculiarities of the Kenyan flow data on the Namibian data (flow symmetry, missing within-unit trip counts, reparametrising the scaling parameter kappa) causes the log-likelihood and several parameters to change substantially (see Table 23 in supporting S1 Text). More rigorous investigations are necessary to fully comprehend the extent to which changes in flow data influence parameter estimates across spatial scales.

**b) The assumption in the analysis is that the same factors such as population size and distance are driving the heterogeneity of travel for finer spatial units. For example, if X trips occur between two districts (or aggregated administrative unit), then the assumption is that the number of trips (that will sum to X) that occur from the grid cells comprising those districts are also driven by population size and distance factors. Since trip data on finer spatial units is unavailable to directly fit parameter estimates, it is unclear if these factors or other factors are the most relevant data sets.**

**This is indeed a relevant observation and underpins the whole analysis. We have highlighted this in the discussion by adding the following text (lines 196-200):**

The assumption underpinning this analysis, is that large scale human travel is driven by the same mechanisms as small scale travel. In other words, we are assuming that metrics on population distribution are sufficient to describe travel propensity at all spatial scales. A lack of very fine scale travel data make it impossible to verify this assumption empirically.

**c) The authors should provide additional support and analyses of the model fits. Currently, enough detail is not provided. In particular, a more thorough comparison and investigation of the estimated model fits versus the data should be provided since it forms the primary result.**

**We agree with the reviewer that this aspect deserves more consideration. We found a bug in the code that created the figure comparing the cumulative frequency of trips by distance of empirical and estimated trip counts in a figure we had previously submitted. Correcting this mistake, revealed that the simulations compare somewhat less well to the empirical data than we had previously thought (Fig 3A). This is confirmed in Table 2 in the supporting S1 Text that shows that at most about 6% of origin-destination flow counts fall within the 95% credible intervals of the simulated flows. However, the overall trip frequency by distance of empirical and simulated data compares well (Fig 3B-D, Table 17 in supporting S1 Text) and proves the value in mathematical mobility models.**

**d) In general, the conclusions do not appear to be the same for Kenya and Namibia. They find opposite results in Kenya and Namibia but do not discuss the implications of these findings further.**

**Thanks for highlighting the paucity in our arguments here. We have commented on this throughout the discussion of the parameter estimates for each country and model, and mentioned it again in the following paragraph (lines 227-234):**

The dissimilarity between the parameter estimates of Kenya and Namibia raises the question of whether population density is a major factor on model fit. However, even restricting the Namibian flow dataset to administrative regions in the more densely populated north does not yield log-likelihoods that improve with spatial resolution or parameter estimates close to the Kenyan ones (Tables 5 and 21 in supporting S1 Text). Hence, the difference might be due to the different methods by which flow counts have been extracted from the CDR datasets of the two countries (see Methods for details).

**e) And in particular, the finer spatial scale models do not perform better in Namibia. The authors should provide additional analyses and interpretation of this finding. Further, in the discussion the authors discuss the sparseness of the population distribution on finer scales. It should be possible to analyze this hypothesis further to provide quantitative support. It is unfair to state in the abstract and results that the finer-spatial models outperformed the aggregate models.**

**Apologies for the unclear formulation. The administrative unit scale clearly performs better than the grid scales in Namibia. We have removed the sentence that made this misleading claim from the abstract. We have addressed the second part of the question with a new set of sensitivity analyses** (Table 21 in supporting S1 Text). **Unfortunately, the findings were not conclusive, which brought us to postulate an influence of the input dataset. We have added the following paragraph in the discussion (lines 227-234):**

The dissimilarity between the parameter estimates of Kenya and Namibia raises the question of whether population density is a major factor on model fit. However, even restricting the Namibian flow dataset to administrative regions in the more densely populated north does not yield log-likelihoods that improve with spatial resolution or parameter estimates close to the Kenyan ones (Tables 5 and 21 in supporting S1 Text). Hence, the difference might be due to the different methods by which flow counts have been extracted from the CDR datasets of the two countries (see Methods for details).

**f) The authors do not provide any interpretation of the model parameters or the differences on various spatial scales. Given that this is the one of the primary results of the manuscript, additional analyses and interpretation should be provided.**

**A review of parameter estimates and their empirical meaning is provided in the discussion on lines 141-190. To aid understanding, we added a lookup table and a figure in** Section 1 of the supporting S1 Text.

**g) The authors do not provide any implications for biological applications. Although the overall motivation is to study infectious disease dynamics, they do not provide any results that evaluate the impact of different models on disease dynamics. For example, the radiation model may outperform the gravity model in some instances, however these differences may not be relevant for disease transmission given the life history of the pathogen.**

**We agree with the reviewer that mobility models should also be compared when applied to infectious disease modelling. However, this aim is outside the scope of this project. For examples from the literature see e.g. (1) for an application of radiation models on the spread of an influenza-like illness, (2,3) for the application of gravity models on influenza transmission, (4) for an application of gravity models on dengue transmission, (5) for the application of gravity models to the spread of measles.**

**h) In the gravity model, the authors have added additional parameters (such as 10^kappa – gamma*epsilon) but do not provide additional support or interpretation for the added these parameters.**

**We agree with the reviewer that it is important to include the explanation for this. We only applied the transformation from 10^kappa to 10^(kappa - gamma * epsilon) to the Kenyan gravity model, and it has to do with the fact that the Kenyan flow data does not give information on within-unit travel. See lines 336-345 for an explanation using mathematical formulae. See Figure 12 in the supporting S1 Text for a visual explanation.**

Minor Comments:

**a) The authors should provide higher resolution figures since the current versions, although interpretable, are blurry to view.**

**Apologies. We added better quality figures.**

**b) There are a number of typos in the manuscript particularly regarding verb tense.**

**Apologies for this too. We hope we have corrected all orthographic mistakes.**

**c) It is unclear exactly how the bottom row of figures in Figure S3 was calculated.**

**We agree that the caption was not very clear. We have considerably expanded the caption. See Figure 3 in the supporting S1 Text.**

**d) The authors should provide code to reproduce their analysis and the predicted model output.**

The code is now available at [**https://github.com/ConniCia/RP01_spatial_fit_code**](https://github.com/ConniCia/RP01_spatial_fit_code)

Reviewer #2:

**In the proposed article, the authors fit different models (gravity and radiation models) to CDR data at different levels of spatial resolution using datasets from Kenya and Namibia. The suitability of often-used gravity models is only partially understood and appears to depend on spatial resolution and across locations. This paper provides an approach to explore these questions. I only have a few comments.

The appropriateness of the different model formulations depends on the quality of the CDR data used to fit the models. This appears particularly problematic for the Kenyan dataset, as there was no direction data available for the flows. The authors come up with an approach that sums over both directions, however, the uncertainty generated by such an approach was not clear to me. As a sensitivity analysis, the authors could aggregate the Namibian dataset to make an equivalent to the Kenyan one (ie with no direction information) and use the same approach. This would at least provide some insight as to the consistency of the results and the potential pitfalls of losing direction information.**

We thank the review for the idea for this sensitivity analysis. We have summarised our findings in Table 23 of the supplementary S1 Text and have added the following text on lines 201-208 of the main text:

Radiation models do not estimate within-unit trips, and the Kenyan dataset does not provide data on within-unit travel. Hence, we were only able to fit the gravity model to Namibian within-unit trip counts. This also applies to trips between cells belonging to the same unit at the grid scales.

Recreating the peculiarities of the Kenyan flow data on the Namibian data (flow symmetry, missing within-unit trip counts, reparametrising the scaling parameter kappa) causes the log-likelihood and several parameters to change substantially (see Table 23 in supporting S1 Text). More rigorous investigations are necessary to fully comprehend the extent to which changes in flow data influence parameter estimates across spatial scales.

**It was unclear how the authors dealt with within-unit travel – in particular, what distance did they use in the gravity models (e.g., distance of 0, 0.5 x cell width, population weighted average based on LandScan……)? – I could imagine this could be quite important to model fit as most travel with be within the same unit.**

**Within-cell travel was assumed to have distance 0. The distance of travel between cells belonging to the same administrative units has been computed using the Euclidean distance, like for all other cell pairs.**

**It was difficult to assess model fit – the summary measures do not explore the fit of individual district pairs. The authors could plot the observed number of trips between each district pair with that predicted from the different models (aggregating the smaller units where necessary). That would allow readers to assess whether there are any systematic differences.**

**We agree with the reviewer that this aspect deserves more consideration. We found a bug in the code that created the figure comparing the cumulative frequency of trips by distance of empirical and estimated trip counts in a figure we had previously submitted. Correcting this mistake, revealed that the simulations compare somewhat less well to the empirical data than we had previously thought (Fig 3A). This is confirmed in Table 2 in the supporting S1 Text that shows that at most about 6% of origin-destination flow counts fall within the 95% credible intervals of the simulated flows. However, the overall trip frequency by distance of empirical and simulated data compares well (Fig 3B-D, Table 17 in supporting S1 Text) and proves the value in mathematical mobility models.**

**Relatedly, it would also be good to understand the robustness of the fit to held-out datasets (e.g., fitting in one part of the country and estimating in another)**

**We thank the reviewer for this exciting idea which we readily implemented.** We have summarised our findings in Table 22 of the supplementary S1 Text **and have inserted the following paragraph in the discussion on lines 209-217:**

Travel data might at times be incomplete, i.e. only detailing trip counts for a selection of travel routes. Sampling 15 random locations from the Kenyan and Namibian dataset, we achieve satisfying fits in terms of log-likelihood from the gravity model (see Table 22 in supporting S1 Text); less so from the radiation model RM4. Increasing the sample size to 30 or even 50 locations considerably improves the log-likelihood. However, parameter estimates are less stable, questioning the ability of mobility models to generalise from a small dataset to a large one (e.g. estimates for alpha and beta for the GM of Kenya in Table 22 in supporting S1 Text). If the list of travel locations is short, there's an increased risk of over-fitting the data.

1.	Tizzoni M, Bajardi P, Decuyper A, Kon Kam King G, Schneider CM, Blondel VD, et al. On the Use of Human Mobility Proxies for Modeling Epidemics. Salathé M, editor. PLOS Comput Biol [Internet]. 2014;10(7):e1003716. Available from: http://dx.plos.org/10.1371/journal.pcbi.1003716

2.	Kissler SM, Gog JR, Viboud C, Charu V, Bjørnstad ON, Simonsen L, et al. Geographic transmission hubs of the 2009 influenza pandemic in the United States. Epidemics [Internet]. 2019;26(August 2017):86–94. Available from: https://doi.org/10.1016/j.epidem.2018.10.002

3.	Truscott J, Ferguson NM. Evaluating the Adequacy of Gravity Models as a Description of Human Mobility for Epidemic Modelling. Pascual M, editor. PLOS Comput Biol [Internet]. 2012;8(10):e1002699. Available from: https://dx.plos.org/10.1371/journal.pcbi.1002699

4.	Wesolowski A, Qureshi T, Boni MF, Sundsøy PR, Johansson MA, Rasheed SB, et al. Impact of human mobility on the emergence of dengue epidemics in Pakistan. Proc Natl Acad Sci U S A [Internet]. 2015;112(38):11887–92. Available from: http://www.ncbi.nlm.nih.gov/pubmed/26351662

5.	Xia Y, Bjørnstad ON, Grenfell BT. Measles Metapopulation Dynamics: A Gravity Model for Epidemiological Coupling and Dynamics. Am Nat [Internet]. 2004;164(2):267–81. Available from: http://www.journals.uchicago.edu/doi/10.1086/422341

6.	Marshall JM, Wu SL, Sanchez C. HM, Kiware SS, Ndhlovu M, Ouédraogo AL, et al. Mathematical models of human mobility of relevance to malaria transmission in Africa. Sci Rep [Internet]. 2018;8(1):7713. Available from: https://www.nature.com/articles/s41598-018-26023-1.pdf
